# Micro-RNA-186-5p inhibition attenuates proliferation, anchorage independent growth and invasion in metastatic prostate cancer cells

**DOI:** 10.1186/s12885-018-4258-0

**Published:** 2018-04-13

**Authors:** Dominique Z. Jones, M. Lee Schmidt, Suman Suman, Katharine R. Hobbing, Shirish S. Barve, Leila Gobejishvili, Guy Brock, Carolyn M. Klinge, Shesh N. Rai, Jong Park, Geoffrey J. Clark, Rajesh Agarwal, LaCreis R. Kidd

**Affiliations:** 1 Department of Pharmacology and Toxicology, University of Louisville School of Medicine, Louisville, KY 40292 USA; 2 James Graham Brown Cancer Center, University of Louisville School of Medicine, Louisville, USA; 3 Department of Pharmaceutical Sciences, Skaggs School of Pharmacy and Pharmaceutical Sciences, University of Colorado, Denver, USA; 4 Division of Gastroenterology and Hepatology, University of Louisville School of Medicine, Louisville, USA; 5 Department of Biomedical Informatics, The Ohio State University, Columbus, USA; 6 Department of Biochemistry and Molecular Genetics, University of Louisville School of Medicine, Louisville, USA; 7 Department of Bioinformatics and Biostatistics, University of Louisville School of Public Health and Information Science, Louisville, USA; 8 Department of Cancer Epidemiology, H. Lee Moffitt Cancer Center and Research Institute, Tampa, USA

**Keywords:** microRNA, Prostate cancer, miR-186, Serum, AKAP12, β-catenin, Metastasis

## Abstract

**Background:**

Dysregulation of microRNA (miRNA) expression is associated with hallmarks of aggressive tumor phenotypes, e.g., enhanced cell growth, proliferation, invasion, and anchorage independent growth in prostate cancer (PCa).

**Methods:**

Serum-based miRNA profiling involved 15 men diagnosed with non-metastatic (stage I, III) and metastatic (stage IV) PCa and five age-matched disease-free men using miRNA arrays with select targets confirmed by quantitative real-time PCR (qRT-PCR). The effect of miR-186-5p inhibition or ectopic expression on cellular behavior of PCa cells *(*i.e., PC-3, MDA-PCa-2b, and LNCaP) involved the use bromodeoxyuridine (BrdU) incorporation, invasion, and colony formation assays. Assessment of the impact of miR-186-5p inhibition or overexpression on selected targets entailed microarray analysis, qRT-PCR, and/or western blots. Statistical evaluation used the modified t-test and ANOVA analysis.

**Results:**

MiR-186-5p was upregulated in serum from PCa patients and metastatic PCa cell lines (i.e., PC-3, MDA-PCa-2b, LNCaP) compared to serum from disease-free individuals or a normal prostate epithelial cell line (RWPE1), respectively. Inhibition of miR-186-5p reduced cell proliferation, invasion, and anchorage-independent growth of PC-3 and/or MDA-PCa-2b PCa cells. AKAP12, a tumor suppressor target of miR-186-5p, was upregulated in PC-3 and MDA-PCa-2b cells transfected with a miR-186-5p inhibitor. Conversely, ectopic miR-186-5p expression in HEK 293 T cells decreased AKAP12 expression by 30%. Both pAKT and β-catenin levels were down-regulated in miR-186-5p inhibited PCa cells.

**Conclusions:**

Our findings suggest miR-186-5p plays an oncogenic role in PCa. Inhibition of miR-186-5p reduced PCa cell proliferation and invasion as well as increased AKAP12 expression. Future studies should explore whether miR-186-5p may serve as a candidate prognostic indicator and a therapeutic target for the treatment of aggressive prostate cancer.

**Electronic supplementary material:**

The online version of this article (10.1186/s12885-018-4258-0) contains supplementary material, which is available to authorized users.

## Background

Prostate cancer (PCa) is the leading cause of non-melanoma cancer-related mortality in men in the U.S. [1]. Ninety-one percent of PCa cases are treatable among men diagnosed with localized or regional disease as evidenced by a 100% 5-year survival rate [1]. However, the 5-year survival rate drops to 28% for those with metastatic PCa [2]. Although early detection of PCa has improved, better prognostic biomarkers are urgently needed to refine current detection, prognosis, and clinical management strategies for metastatic PCa [3].

MicroRNAs (miRs or miRNAs) are 17–25 nucleotide short non-coding RNAs that may serve as ideal biomarkers of metastatic PCa for several reasons. First, miRs are stably expressed in tumor tissue and related biological fluids, including serum and plasma [4]. Second, miRs regulate the expression of genes involved in tumor spread including cell proliferation, invasion, migration, angiogenesis, and anchorage-independent growth (reviewed in [5–7]). Third, dysregulation of miRNA corresponds with aggressive PCa phenotypes including high tumor stage, high Gleason grade, disease recurrence, and biochemical recurrence [8, 9]. Lastly, serum miRNA profiles may distinguish between aggressive and non-aggressive PCa [10, 11].

Dysregulation of miRNAs is associated with physiological changes in tumorigenesis and disease progression in PCa (reviewed in [12]). The role of miR-186 is cancer type-specific and has oncogenic or tumor suppressor roles. For example, miR-186 expression is upregulated in melanoma, endometrial, pancreatic, esophageal, and cervical cancers, suggesting an oncogenic role in these cancers [13–19]. The predominant form of miR-186 is miR-186-5p. The influence of miR-186 expression on tumor cellular behavior (i.e., proliferation, invasion, and anchorage independent growth) has been evaluated in pancreatic [17], bladder [20], ovarian [21], and non-small cell lung cancer (NSCLC) cells [22–27]. MiR-186 acts as a tumor suppressor in NSCLC, since its overexpression reduced cell invasion and migration of NSCLC cell lines in vitro [23, 24, 26]. In contrast, overexpression of miR-186 in pancreatic [17] and bladder [20] cancer cells enhanced cellular proliferation, migration, colony formation and anchorage independent growth, implicating an oncogenic role for miR-186 in these cancers. Overexpression of miR-186 also repressed the expression of tumor suppressors, including forkhead box O1 (*FOXO1*), Nuclear Receptor Subfamily 5 Group A Member 2 (*NR5A2*), and protein phosphatase, Mg^2+^/Mn^2+^ dependent 1B (*PPM1B*) in endometrial [14], pancreatic [17], and bladder [20] cancer cells, respectively. However, the functional role of miR-186-5p and its targets in PCa remains unclear [28–32]. One report suggested miR-186 functions as a tumor suppressor miRNA in PCa [30]. The authors observed a down-regulation of miR-186 in five human PCa cell lines versus primary cultured prostate epithelial cells and tumor tissue compared with adjacent normal prostate. However, it is not clear whether they evaluated miR-186-3p or miR-186-5p. Moreover, to our knowledge, there are no published reports on the evaluation of miR-186-5p in PCa patient serum.

The purpose of the current study was to identify differentially expressed miRNAs in serum from PCa patients versus normal controls. In addition, this study sought to characterize the role of miR-186-5p in metastatic PCa cell models. Our findings will aid in the understanding of miR-186-5p’s role in metastatic PCa using pre-clinical, human PCa cell lines. Our novel identification of increased miR-186-5p in the serum of metastatic PCa patients and its pro-migration/invasion oncogenic activity in PCa cell lines suggests miR-186-5p may serve as a diagnostic, prognostic and ultimately a therapeutic tool for the effective clinical management of metastatic PCa.

## Methods

### Human serum biospecimens

Serum samples (0.5–1 ml) were collected from five disease-free controls and 15 men diagnosed with PCa prior to any therapy. All specimens were obtained from BioServe Biotechnologies Biorepository (Beltsville, MD). PCa patients were diagnosed with tumor stage I (*n* = 5), stage III (*n* = 5), and stage IV (*n* = 5) disease. De-identified demographic and clinico-pathological data for each patient included age, weight, and body mass index (BMI). There was no follow-up data available for these patients after treatment. Samples were stored at -80 °C until further use.

### Cell culture

Human prostate cancer metastatic [PC-3 (ATCC CRL-1435), DU145 (ATCC HTB-81), LNCaP (ATCC CRL-1740), 22Rv1 (ATCC CRL-2505), MDA-PCa-2b (ATCC CRL-2422)], embryonic kidney HEK 293 T (ATCC CRL-3216) and normal prostatic epithelial [RWPE1 (ATCC CRL-11609), RWPE2 (ATCC CRL-11610)] cell lines were obtained from American Type Culture Collection (ATCC) (Manassas, VA). Prostate cancer cell lines were cultured in Dulbecco’s Modified Eagle’s Media (HEK 293 T, DU145), RPMI 1640 (22Rv1, LNCaP), T-media (C4-2B) [33], and Kaighn’s modified Ham’s F-12 K media (PC3). MDA-PCa-2b cells were grown in F-12 K media supplemented with hydrocortisone (100 pg/ml), EGF (10 ng/ml), and FBS (20%). Normal prostate epithelial cells (RWPE1, RWPE2) were grown in Keratinocyte-SFM supplemented with bovine pituitary extract (BPE) 50 μg/ml) and human recombinant epidermal growth factor (EGF) (5 ng/ml). All media was supplemented with 10% of FBS and 1% of antibiotic [10,000 I.U./ml of penicillin, 10,000 μg/ml of Streptomycin, 25 μg/ml Amphoterricin B}.

### Isolation of miRNAs from serum

Serum (250 μl) obtained from 15 patients and five disease-free individuals was transferred to 1.5 ml nuclease-free tubes. Trizol LS Reagent (1 ml) was added to each sample and shaken for 30 s (secs) at room temperature. Each sample was spiked with cel-miR-39 (2 μl, 1 nM, internal miRNA control) and incubated for 5 min (mins). ACS 98% grade chloroform (200 μl per 1 ml of Trizol) was added to each sample, shaken for 15 s, and incubated again for 5 mins. Next, total RNA was isolated from serum using the miRVana microRNA Isolation kit (Thermo Fisher Scientific, Waltham, MA).

### miR profiling in serum using Taqman human MicroRNA arrays

Expression analysis of 377 miRNAs involved the use of Taqman Array Human MicroRNA Pool A Cards v.2 (Thermo Fisher Scientific) that included three endogenous controls (RNU6, RNU44, RNU48) and a non-human related negative control (ath-miR-159a). Micro-RNA profiling was assessed using the Applied Biosystems 7900 Real Time PCR system (Thermo Fisher Scientific). RNA was reverse transcribed into cDNA in a 7.5 μl reaction using a TaqMan miRNA Reverse Transcription (RT) Kit and MegaPlex RT Primers (10X) (Thermo Fisher Scientific). Diluted pre-amplified RT products in TE (75 μl, 0.1X, pH 8.0) were added to a reaction mix [100 μl of Taqman Universal PCR Master Mix (2X)] and dispensed into arrays. miRNA profiles in serum were normalized to the global median comparative threshold (Ct) value for each array (global Ct median value - target Ct value) using R-programming software. Fold change was calculated with respect to each tumor stage relative to disease-free individuals. After normalization, targets with ≥50% missing Ct values were imputed using k nearest neighbor (kNN) imputation. Differentially expressed serum-based miRNAs in PCa patients were selected for further validation using the following selection criteria: FDR *p*-value ≤0.05 and fold change ≥1.5.

### RNA/microRNA isolation from cells and qRT-PCR

Total microRNA was extracted and purified using the miRVana miRNA Isolation kit according to manufacturer’s instructions. RNA (5 μl) was converted into cDNA using the MicroRNA Reverse Transcription (RT) kit and specific RT primers (Thermofisher Scientific). Total RNA (500 ng or 1 μg) was reverse transcribed into cDNA using qScriptTM cDNA SuperMix (Quanta Biosciences, Beverly, MA). cDNA from cells was mixed with PerfeCTa SYBR Green FastMix ROX (Quanta Biosciences, Beverly, MA) or Taqman Universal Mix II No UNG plus specific PCR primers; AKAP12 (Qiagen, Germantown, MD), and TaqMan Assays [miR-106b-5p (Assay # 000442), miR-302b-3p, (Assay # 000531), miR-520e (Assay # 001119), miR-342-3p (Assay # 002260), miR-186-5p (Assay # 002285), miR-885-5p (Assay # 002296)] for qPCR using the Step Up Real PCR system (Applied Biosystems, Waltham, MA). Relative expression of mRNA and miRNA was normalized to GAPDH and snoRNA U44 expression and calculated using the 2-∆∆Ct method. Experiments were repeated three times and in triplicate.

### Validation of serum microRNAs using qRT-PCR

miRNAs were evaluated within the same cohort of PCa serum samples using Taqman RT and PCR assays for miRs-106b-5p, −302b-3p, −520e, − 342-3p, − 186-5p, and − 885-5p (Thermo Fisher Scientific). miRNA was detected in the RNA/microRNA Isolation from cells and qRT-PCR sections, as previously described. Relative miRNA expression was calculated using the 2^-∆∆Ct^ method and normalized to spiked-in miR-cel-39 (serum).

### DNA isolation from cells

RWPE1 cells were grown to 80% confluency in growth media. Genomic DNA was isolated from cells using DNeasy Blood and Tissue kit (Cat# 69504, Qiagen, Valencia, CA). DNA concentrations (260/280 nm) were measured using a Nano Dropper Spectrophotometer.

### Plasmid constructs

To construct the pcDNA-DEST47-miR-186 mimic, the full-length of the miR-186-5p precursor was amplified from human genomic DNA and cloned into the pENTR/D-Topo vector (ThermoFisher Scientific) with BamHI and NotI restriction enzymes. The primer sequences for miR-186-5p were: miR-186-5p forward (5’-GCggatccGAGCCATGCTTATGCTACTG-3′) and miR-186-5p reverse (5′ -GCgcggccgcCCAGGTATATGGCA-3′).

To construct the pcDNA-DEST47-anti-miR-186, the full-length of anti-sense miR-186-5p amplified from human genomic DNA of RWPE1 cells was cloned into the pENTR/D-Topo vector (Thermo Fisher Scientific) and shuttled into pcDNA-DEST47 mammalian expression vector with BamHI and NotI restriction enzymes. The anti-sense oligonucleotide sequences were: anti-miR-186-5p forward (5’ CACCGCggatccTGCTTGTAACTTTCCAAAGAATTCTCTCCTTTTGGGCTTTCTGGTTTTATTTTAAGCCCAAAGGTGAATTTTTTGGGAAGTTTGAGCT-3′) and anti-miR-186-5p reverse (5’ gcggccGCAGCTCAAACTTCCCAAAAAATTCACCTTTGGGCTTAAAATAAAACCAGAAAGCCCAAAAGGAGAGAATTCTTTGGAAAGTTACAAGCA-3′). Clones were verified via DNA sequencing by Eurofins Genomics (Louisville, KY).

### miRNA mimic and inhibitor transfection

Biological effects of aberrant miR-186-5p expression were studied by both stable and transient transfection of pcDNA-DEST47 constructs (1 μg pcDNA-DEST47-miR-186-5p, pcDNA-DEST47-anti-miR-186-5p), mimic (33 nM Assay# MC11753, Cat# 4464066) and inhibitor (33 nM, Assay# MH11753, Cat# 4464084) and respective negative controls (pcDNA-DEST47, NC-mimic Cat# 4464078, NC-inhibitor Cat# 4464078) from ThermoFisher Scientific in PC-3, MDA-PCa-2b, LNCaP, RWPE1, and HEK 293 T cells. Cells were seeded in 60 mm dishes and transfected in Opti-MEM reduced serum media using JetPrime reagent (Polyplus Transfection, New York, NY) and/or Superfect reagent (Catalog# 301305, Qiagen, Valencia, CA) according to manufacturer’s instructions, respectively. Stably transfected cells were selected using growth medium containing 800 μg/ml G418 Sulfate. Stable transfected cell clones were maintained and passaged in culture medium with G418 (400 μg/ml). Cells were harvested for cellular behavior assays (i.e., cellular proliferation, invasion, and colony formation assay) 24 h post-transfection. Ectopic expression and inhibition of miR-186-5p in total RNA and whole cell protein lysate were confirmed via qRT-PCR.

### Human gene expression array

RNA was extracted from transient and stable transfected PC-3 cells with miR-186-5p inhibitor, RWPE1 cells with miR-186-5p mimic, and corresponding scramble (transient) or empty vector controls (stable) in 3 independent experiments. RNA sample purity and integrity were assessed using the Agilent 2100 Bioanalyzer. RNA (250 ng) was serial diluted and transcribed into cDNA and cRNA. Fragmented and biotin-labeled cRNA (12 μg) was subjected to a series of incubation periods and hybridized to Prime View gene microarrays with appropriate poly-A and hybridization controls using the 3’IVT Plus Reagent kit (Cat# 902416, Affymetrix Inc., Santa Clara, CA), according to the manufacturer’s instructions. Each array was washed and stained according to array type. The fluidics protocol FS450_0002 was used to analyze each array via Gene chip scanner (Affy.Command console Version 3.3).

### Gene selection

Aberrant gene expression associated with miR-186-5p modification was identified via microarray analysis. Genes up-regulated in stable miR-186-5p inhibited PC-3 cells and down-regulated in stable miR-186-5p overexpressing RWPE1 cells were identified as potential miR-186-5p targets (± 1.2 fold change in expression and false discovery rate *p*-value < 0.05). Next, potential targets were evaluated based on published reports and *in silico* databases, including MetaCore, Ingenuity, www.TargetScan.org and the www.microrna.org. Final selection of miR-186-5p targets was based on published reports and www.microrna.org database. The aforementioned miR database used PhastCons (positive value ≥0.57) and mirSVR (negative score ≤ − 0.1) scoring methods to determine highly conserved miRNAs [12, 34].

### BrdU proliferation assay

Cell proliferation in cells was measured using the Cell Proliferation ELISA 5-bromo-2′-deoxyuridine (BrdU) colorimetric kit (#11647229001, Sigma Aldrich, St. Louis, MO). Transfected cells (5 × 10^3^/well) were seeded into a 96-well plate format, incubated at 37 °C for 24 h and labeled with BrdU reagent for 24 h. Absorbance readings were taken at 370 nm and 492 nm (reference) using a Biotek Synergy HT plate reader and Gen5 version 1.08 software (BioTrek, Winooski, VT). Experiments included experimental groups with six replicates that were repeated at least three times.

### Anchorage-independent growth assay

The influence of ectopic expression and inhibition of miR-186-5p on 2-dimensional colony formation was assessed using an anchorage independent growth assay. In 6-well plates, 0.7% agar-growth media solution (3 ml), prepared with sterile 3.5% agar and 1X phosphate buffered saline (PBS), was added to each well to form a base layer. Transfected cells (10 × 10^3^) in growth media (3 ml) were gently mixed with 0.7% agar-media solution (3 ml) seeded on top of base layers. Cells in soft agar were incubated at 37 °C for 2–3 weeks. Colonies were quantitated at 4X magnification. Experiments were repeated at least three times.

### Matrigel invasion assay

The effect of miR-186-5p inhibition on cellular invasion was evaluated by the Boyden chamber assay, as described elsewhere (Albini,A. et al. 1987). Briefly, polyethylene transwell inserts with 8 μm pore size were coated with a final concentration of 2 mg/ml of reduced growth matrigel. Cells (25 × 10^3^) were suspended in serum-free media containing reduced growth Matrigel and seeded on top of matrigel. Growth media with FBS (600 μl) was added to the lower chamber of each well. After 24 h of incubation (37 °C, 5% CO2), non-invading cells on the upper side of the membrane were removed with 1X PBS. Invading cells were fixed in 100% methanol and stained with 0.2% crystal violet. The number of invading cells was counted under a microscope (EVOS) quantified using a 10X magnification. Assays were repeated at least three times.

### Western blot analysis

Whole cell protein lysates were collected from transiently transfected HEK 293 T, MDA-PCa-2b and PC-3 cells 24–96 h post-transfection using Radio-Immunoprecipitation Assay (RIPA) buffer (Cat #R0278, Sigma Aldrich, St. Louis, MO) supplemented with 100 mM sodium orthovanadate and protease inhibitor cocktail (Sigma Aldrich). Protein concentrations were determined using Bradford’s assay (Bio-Rad, Hercules, CA). Samples (35 or 45 μg) were separated by MP TGX 4–20% gels and transferred to PVDF membranes using the Trans-Blot Turbo system (Bio-Rad). Membranes were blocked in 5% milk for 1 h. AKAP12, β-catenin, and phospho-AKT were measured using primary monoclonal mouse AKAP12 antibody (1:500, Sigma Aldrich), primary mouse β-catenin antibody (1:1000, Cell Signaling, Danvers, MA), monoclonal rabbit phospho-AKT (Ser473) (1:1000, Cell Signaling), secondary anti-mouse antibody (1:10,000, Cell Signaling), secondary anti-rabbit antibody (1:20,000, Cell Signaling) and β-actin (1:5000, Cell Signaling) as a loading control. Densitometry analysis was performed using ImageJ software (U. S. NIH, Bethesda, MD). Experiments were repeated 2–3 times.

### Statistical analysis

Differences in demographic/clinical data [age, prostate specific antigen (PSA) levels and BMI values] comparing PCa patients and controls were assessed using the Wilcoxon Rank-Sum test. Differential miRNA expression for each tumor stage was adjusted for multiple hypothesis testing (i.e., FDR) relative to non-cancerous controls using ANOVA and modified t-test with the R package limma [35, 36]. Differential gene expression was identified in PC-3 and RWPE1 cells using the Partek Genomics Suite 6.6 software (St. Louis, MO), after adjusting for multiple hypothesis testing using the false discovery test (FDR). MicroRNA/mRNA expression and biological assays were evaluated using two-sided unpaired t-tests. (GraphPad 6 Software, Inc., La Jolla, CA). All statistical significance was established using an alpha cut-off value of 0.05 or FDR ≤ 0.05. All statistical analysis was performed using GraphPad 6 Software, Inc., (La Jolla, CA).

## Results

### Population description

Serum was collected from 15 PCa patients diagnosed with tumor stage I, III, IV and five disease-free patients who self-identified as men with European ancestry (Additional file 1: Table S1). There was no significant difference in the median age or BMI levels between cases and controls, respectively. Median PSA levels among cases were significantly higher than non-cancerous controls (*p* = 0.048). Notably PSA levels in controls were higher than the normal range. According to the clinical data provided for the control biospecimens, each patient was classified as having a normal prostate at the time of serum collection. Although the “control” subjects had high PSA levels, their prostate was designated as disease-free or “normal” based on negative biopsy results. Unfortunately, there was no data on whether the controls had BPH and there was no available clinical follow-up data for the patients designated as controls. Tumor classification for 60 % (*n* = 9) of the cases were diagnosed with adenocarcinoma and 67% had a smoking history. However, the smoking history status among the controls was not available for this study population. The majority of the PCa patients received at least two types of therapy (73.3%), including hormonal therapy (*n* = 14, 93.3%), radiation (*n* = 8, 53.3%), surgery (*n* = 7, 46.7%), and chemotherapy (*n* = 2, 13.3%).

### Differentially expressed miRNAs in the serum of PCa patients

We evaluated the expression of 377 miRNAs in the serum from 15 PCa patients diagnosed with tumor stage I (*n* = 5), III (*n* = 5) and IV (*n* = 5) compared to disease-free individuals (*n* = 5). Twenty-six miRNAs were differentially expressed in the serum from PCa patients (*p* ≤ 0.05, fold change ≥1.5 or ≤ − 1.5). After adjusting for multiple hypothesis testing, we selected 6 miRs for validation, namely, miRs-106b-5p, − 186-5p, −302b-3p, − 342-3p, −520e, and − 885-5p (FDR *p*-value ≤0.05), as shown in Additional file 2: Table S2. Two miRs (−106b and − 186) were validated by qRT-PCR. However, we focused on miR-186-5p (FDR *p*-value = 0.005) since there were 22 studies on miR-106b and prostate cancer but only 4 published reports on the role of miR-186 in PCa [29, 30, 32, 37].

### Validation of miR-186-5p in PCa patient serum

**Fig. 1:**
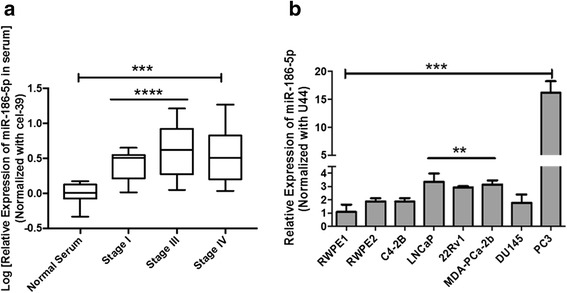
MiR-186-5p expression in PCa serum and cell lines**. a** Relative expression of miR-186-5p was validated in two independent isolations of miRNA from PCa serum (*n* = 15) relative to non-cancerous controls (*n* = 5). Elevated levels of miR-186-5p were detected in the serum of patients diagnosed with PCa tumor stage I and III (*p* ≤ 0.0001), and IV (*p* = 0.0007) disease. **b** Relative miR-186-5p expression was measured in normal prostate epithelial cells (RWPE1, RWPE2), prostate carcinoma xenograft (22Rv1), bone (PC-3), lymph node (LNCaP), brain (DU145), and LNCaP –derived bone (C4-2B) metastatic PCa cells using qRT-PCR. MiR-186-5p was up-regulated in four metastatic PCa cell lines (LNCaP, MDA PCa-2b, PC-3, 22Rv1) relative to normal prostate epithelial RWPE1 cells (*p* < 0.01). PC-3 cells exhibited the highest expression among the other cell lines (*p* = 0.0002). Data analyses were based on 2–3 independent experiments and presented as log [mean fold change] and mean fold change ± standard deviation (S.D.). (** *p*-value < 0.01, *** *p*-value < 0.0007, **** *p*-value < 0.0001) and one-way ANOVA analysis (** *p*-value < 0.005)

MiR-186-5p expression was validated in two independent experiments within the same cohort of serum samples. MiR-186-5p was significantly upregulated in PCa serum relative to disease-free individuals (Fig. 1a). To examine its potential role in PCa, miR-186-5p was selected for further validation and characterization using PCa cell lines and normal prostate epithelial cells.

### Expression of miR-186-5p in metastatic and non-metastatic PCa cell lines

MiR-186-5p was significantly higher in metastatic PCa cell lines (LNCaP, MDA-PCa-2b and PC-3) relative to the control RWPE1 cell line (Fig. 1b). Interestingly, miR-186-5p expression was highest in metastatic PC-3 cells. However, expression of miR-186-5p did not vary significantly between androgen-sensitive (MDA-PCa-2b, LNCaP, C4-2B) and insensitive (PC-3, DU145) cell lines. miR-186-3p expression was detected at high Ct values in PCa cell lines (data not shown).

### Inhibition of miR-186-5p reduces PCa cell proliferation

**Fig. 2:**
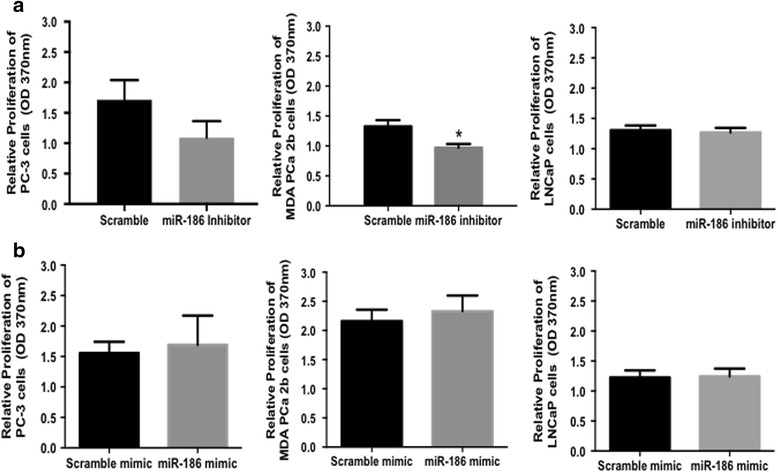
Inhibition of miR-186-5p reduced MDA-PCa-2b PCa cell proliferation. PCa cells were transiently transfected for 24–48 h with miR-186-5p inhibitor, mimic, and scramble negative controls. Cell proliferation was measured by the BrdU assay. **a** MiR-186-5p inhibition resulted in a significant decrease in MDA-PCa -2b (*p* = 0.013) relative to scramble control. **b** Ectopic miR-186-5p expression did not alter proliferation of PC-3 (*p* = 0.6744), MDA PCa2b (*p* = 0.4220) and LNCaP (*p* = 0.8582) relative to scramble control. Data analyses were based on 3 independent experiments and presented as mean absorbance values ± S.D. (*p-value < 0.02)

Since miR-186-5p was upregulated in the serum of the metastatic PCa patients, we evaluated the impact of miR-186-5p inhibition on cell proliferation. If miR-186-5p is oncogenic in PCa cells, we expected inhibition of miR-186-5p would decrease cell proliferation. Indeed, inhibition of miR-186-5p (by transfection of miR-186-5p inhibitor Additional file 3: Figure S1A) reduced cell proliferation by 36% in metastatic MDA-PCa-2b and slightly (27%, non-significant) in PC-3 cells (Fig. 2a). Although miR-186-5p expression was upregulated in LNCaP cells (Fig. 1b), inhibition of miR-186-5p did not affect LNCaP cell proliferation (Fig. 2a, Additional file 3: Figure S1A). In contrast, ectopic expression of miR-186-5p (by transfection of a miR-186-5p mimic, Additional file 3: Figure S1B) did not affect PCa proliferation (Fig. 2b).

### MiR-186-5p modifies anchorage-independent colony formation in metastatic PCa cell lines

**Fig. 3:**
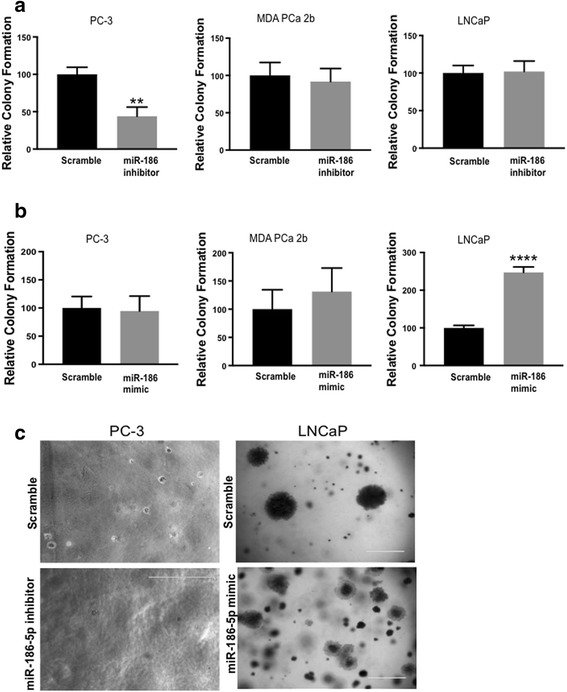
Alteration of miR-186-5p levels affects PCa cell colony formation. Metastatic PC-3 and MDA-PCa-2b cells were transiently transfected with miR-186-5p inhibitor and mimic for 24 h and grown in 0.35% soft agar for 2–3 weeks at 37 °C. **a** Inhibition of miR-186-5p reduced PC-3 cell colony formation (*p* = 0.0033). **b** Ectopic expression of miR-186-5p increased in colony growth in LNCaP cells (*p* < 0.0001). **c** Representative images of anchorage-independent growth of PC-3 and LNCaP cells transfected with miR-186-5p inhibitor or miR-186-5p mimic, respectively. Data analyses were based on at least 3 independent experiments and presented as mean percentage ± S.D. (**p-value < 0.004, ****p-value < 0.0001)

Previous reports demonstrate miR-186 affects anchorage-independent growth in cancer cells [20, 31]. Consequently, we investigated whether inhibition of miR-186-5p alters colony growth of the metastatic PC-3, MDA-PCa-2b, and LNCaP cells. Relative to scramble control, inhibition of miR-186-5p reduced colony formation/anchorage-independent cell growth of PC-3 cells, but not MDA-PCa-2b or LNCaP cells (Fig. 3a, c). Conversely, ectopic expression of miR-186-5p significantly increased colony formation in LNCaP cells, but had no effect on PC-3 or MDA-PCa-2b cells (Fig. 3b, Additional file 3: Figure S1B). These data suggest the impact of miR-186-5p on anchorage-independent cell growth may be saturated in PC-3 cells, perhaps by cell-specific factors that modulate miR-186-5p’s stimulation of anchorage-independent cell growth.

### Suppression of cell invasion in metastatic PCa

**Fig. 4:**
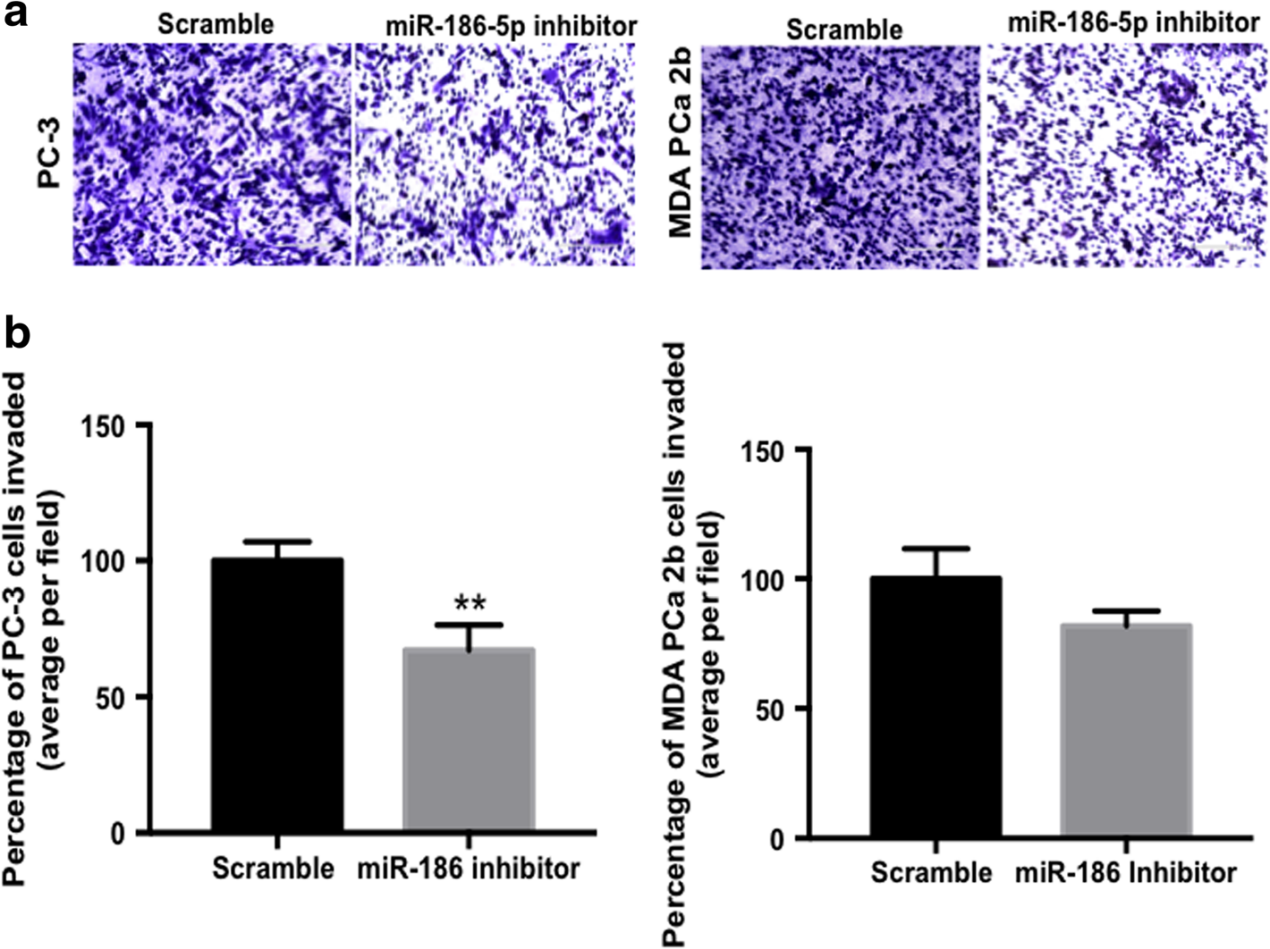
Inhibition of miR-186-5p reduces metastatic PCa cell invasion. PC-3 and MDA-PCa -2b cells were transiently transfected with scrambled control or miR-186-5p inhibitor for 24 h. Post-transfection, cell invasion through the Matrigel-coated filter was assessed using a transwell assay. **a** Representative images of PC-3 and MDA-PCa-2b cells after 24 h, 20X magnification. **b** Quantification of cell invasion showed the miR-186-5p inhibitor reduced PC-3 invasion (*p* = 0.008). Data were quantitated from at least three independent experiments using an average of four fields of view at 10× magnification. Data were presented as mean percentage ± S.D. (**p-value < 0.009)

Since miR-186-5p stimulated anchorage-independent cell growth in PC-3 and LNCaP cells, we evaluated whether inhibition of miR-186-5p affected invasion of metastatic PC-3 and MDA-PCa-2b cells (Figs. 2 and 4a, Additional file 3: Figure S1A). Inhibition of miR-186-5p significantly reduced PC-3 cell invasion, but had no significant effect on MDA-PCa-2b cells (Fig. 4a and b).

### Identification of miR-186-5p gene targets

To identify potential miR-186-5p targets in PCa, gene expression was evaluated in stably miR-186-5p inhibited PC-3 cells and miR-186-5p overexpressed RWPE1 cells (Additional file 3: Figure S1C). After filtering according to the selection criteria [±1.2 fold change, false discovery rate (FDR) *p* ≤ 0.05], microarray analysis identified 1041 direct miR-186-5p targets. Among these potential miR-186-5p targets, 493 were down-regulated in miR-186-5p-overexpressing RWPE1 cells and 547 were upregulated in miR-186-5p-inhibited PC-3 cells (Additional file 4: Table S3). Importantly, four previously validated miR-186-5p targets (e.g., *AKAP12, ROCK1, PPM1B*, and *PTTG1*) were identified using our microarray analysis coupled with *in silico* tools, i.e., miR Base, microRNA.org, Metacore and Ingenuity Pathway Analysis (Additional file 4: Table S3). Direct target selection using a ± 2-fold change cut-off revealed 50 genes (30 targets in PC-3, 20 targets in RWPE1) (Table 1). MiR-186-5p target gene validation was further restricted based on the availability of antibodies for targets. Preference was given to tumor suppressor-related targets based on our results showing oncogenic activity of miR-186-5p in metastatic PCa cell assays (Figs. 2, 3 and 4).

**Table 1:** Identification of potential miR-186 targets in prostate cancer

Cell line	Gene	Fold Change	FDR *p*-value	Cell line	Gene	Fold Change	FDR *p*-value
PC-3	PMEPA1	6.255	8.50219E-12	RWPE1	EHF	−6.261	2.15476E-10
	FN1	5.820	5.69038E-10		ZNF711	−4.141	1.78879E-09
	TSC22D3	4.701	1.20663E-06		GPC6	−3.327	1.39841E-06
	EFEMP1	4.334	3.61878E-07		FOXG1	−2.929	0.000587093
	EGR1	4.130	8.19665E-07		CPE	−2.769	4.23842E-05
	TUBE1	3.931	1.90071E-08		CLCA2	−2.613	0.000051171
	JAG1	3.229	4.83412E-06		CCL20	−2.581	6.13709E-05
	ZNF674	2.983	8.39119E-07		SPX	−2.496	4.34943E-07
	SLITRK6	2.911	6.95917E-06		ZFP42	−2.431	3.0742E-06
	PSAT1	2.587	3.20654E-09		**AKAP12**	−2.376	0.000243387
	KCNT2	2.489	1.49162E-06		ALDH1A2	−2.321	3.93333E-05
	VEGFA	2.489	4.31602E-11		HECTD2	−2.319	0.000018492
	CEBPG	2.472	4.43309E-11		MCTP1	−2.239	1.89756E-07
	KLF9	2.426	7.74733E-06		APOLD1	−2.228	0.000016442
	WNT5A	2.426	2.56651E-07		HMGN5	−2.183	0.000131712
	TRIM36	2.403	7.29682E-07		TPRG1	−2.182	7.81032E-06
	SLC22A15	2.399	3.03846E-05		GPR19	−2.172	3.87728E-06
	CSGALNACT1	2.374	2.85369E-07		BCL11A	−2.145	6.80441E-05
	RHOB	2.328	1.55029E-05		GJA3	−2.036	1.67961E-05
	SAT1	2.291	5.84464E-10		BCL2L11	−2.030	4.64641E-05
	MAML3	2.288	0.000130184				
	ERRFI1	2.200	1.12777E-10				
	KDM7A	2.193	2.32714E-06				
	ZNF558	2.179	0.000232804				
	CD55	2.121	6.2058E-07				
	APOBEC3F	2.112	0.000469815				
	PLEKHG1	2.081	2.30428E-05				
	MTHFD2	2.048	3.7309E-12				
	BCL6	2.039	5.70622E-05				
	C20orf197	2.011	0.000166965				

Statistical significance was established at a 0.05 significance level

### *AKAP12* is a direct target of miR-186-5p

**Fig. 5:**
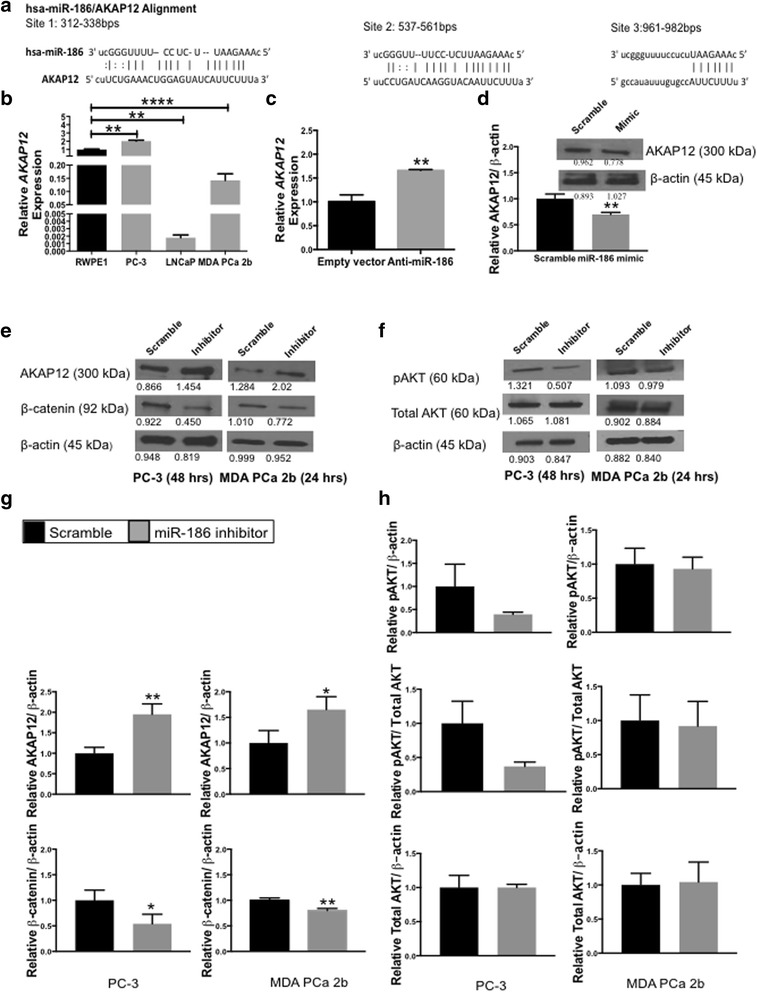
Tumor suppressor AKAP12 is a target of miR-186-5p in PCa cells. AKAP12 transcript and protein expression were measured using qRT-PCR and western blot analysis, respectively. **a** Three potential miR-186-5p binding sites in AKAP12 were identified; site 1) mirSVR score: − 0.5641 PhastCons score: 0.6188; site 2) mirSVR score: − 0.7981 PhastCons score: 0.5553; and site 3) mirSVR score: − 0.2640 PhastCons score: 0.6218 (values from www.microrna.org). **b** *AKAP12* transcript expression was significantly lower in metastatic LNCaP (*p* = 0.0011) and MDA-PCa-2b (*p* < 0.0001) cells and higher in PC-3 cells relative to normal prostate RWPE1 cells (*p* = 0.0011). **c** Stable anti-miR-186-5p expression in PC-3 cells increased *AKAP12* transcript expression (*p* = 0.0013). AKAP12 expression was normalized to GAPDH. **d** HEK 293 T cells were transfected with scrambled miR control or miR-186-5p mimic. A representative western blot shows ectopic expression of miR-186-5p in HEK 293 T cells decreased AKAP12 protein by 30% 72 h post-transfection (*p* = 0.0063). All analyses involved at least three independent experiments. **e** and **f** Representative western blots of AKAP12, β-catenin and pAKT expression in HEK 293 T (72 h), PC-3 (48 h), and MDA-PCa-2b (24 h) cells post-transfection. Data analyses included three independent experiments for **e** and two experiments for **f**. **g** and **h** Quantitation of western blots presented as black bar (scramble control) and grey bar (miR-186-5p inhibitor). AKAP12 protein was increased in PC-3 (*p* = 0.0049) and MDA-PCa-2b (*p* = 0.0318) cells transiently transfected with miR-186-5p inhibitor. In contrast, β-catenin/ β-actin protein was decreased in PC-3 (*p* = 0.0434) and MDA-PCa-2b (*p* = 0.0048) cells transfected with miR-186-5p inhibitor. Data analysis was based on mean ± S.D. of target protein relative to β-actin in the same blot. (*p-value < 0.05, **p-value < 0.007, **** *p*-value < 0.002)

Tumor suppressor targets included A-kinase anchor protein 12 (*AKAP12*), Tumor Protein P53 (p53, *TP53*), Forkhead Box O3 (*FOXO*3), and Phosphatase and Tensin Homolog (*PTEN*) (Additional file 4: Table S3). AKAP12 was selected for validation given its multifaceted role in cell death, cell proliferation, cell invasion, colony formation and epithelial mesenchymal transition [32]. AKAP12 is a scaffolding protein associated with protein kinases A (PKA) and C (PKC). There are three predicted binding sites for miR-186-5p in *AKAP12* (Fig. 5a). The three sites were ranked by mirSVR and PhastCons scoring methods. According to PhastCons scoring, binding sites would be ranked in the following descending order: 3 (Score: 0.6218), 1 (Score: 0.6188) and 2 (Score: 0.5553). However based on mirSVR scoring, the binding sites were ranked in the following ascending order: 2 (Score: − 0.7981), 1(Score: − 0.5641) and 3 (Score: − 0.2640). Relative to RWPE1 normal prostate epithelial cells, *AKAP12* transcript levels were higher in PC-3 cells and lower in LNCaP and MDA-PCa-2b cells (Fig. 5b). We examined whether inhibition of miR-186-5p would increase *AKAP12* transcript levels in PC-3 cells. Indeed, AKAP12 transcript expression increased in miR-186-5p inhibited PC-3 cells (Fig. 5c, Additional file 5: Figure S2A). This observation suggests *AKAP12* is a direct target of miR-186-5p in PC-3 cells.

To further validate AKAP12 as a direct target of miR-186-5p, AKAP12 protein expression was examined in HEK 293 T and PCa cell lysates after transfection with a miR-186-5p mimic or a miR-186-5p inhibitor (Fig. 5d, e, g). However, AKAP12 protein expression was not detected in LNCaP cells. Ectopic miR-186-5p expression reduced AKAP12 protein expression in HEK 293 T cells by 30% (Fig. 5d, Additional file 5: Figure S2B). Inhibition of miR-186-5p increased AKAP12 protein expression by ~ 2-fold in PC-3 and ~ 1.6-fold in MDA-PCa-2b cells (Fig. 5e, g).

### Inhibition of miR-186-5p reduces pAKT and β-catenin

**Fig. 6:**
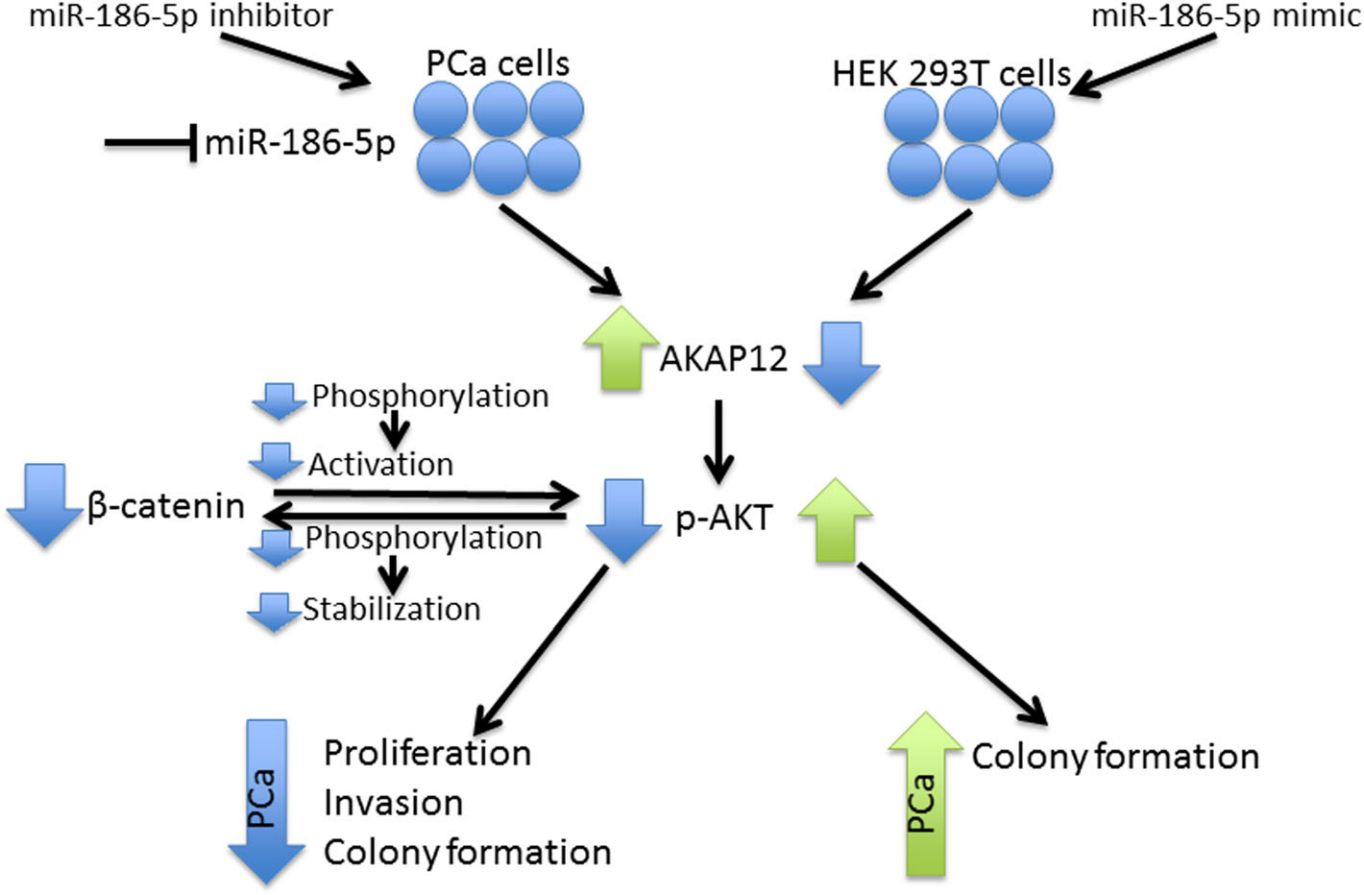
Model of oncogenic miR-186-5p activity in PCa. Transient inhibition of miR-186-5p resulted in an increase in the tumor suppressor AKAP12 and down-regulation of tumorigenic β-catenin in PC-3 cells. Inhibition of miR-186-5p resulted in a reduction in pro-survival p-AKT. However, ectopic miR-186-5p expression corresponded with an elevation in p-AKT

AKAP12 is a molecular scaffold that interacts with PKA (Protein kinase A), PKC (Protein kinase C), tyrosine kinases, and other plasma membrane receptors [32]. Previous studies reported an increase in phospho-AKT (pAKT) was associated with nuclear accumulation of β-catenin (*CTNNB1*) (reviewed in [31, 38]). Further, knockout of *Akap12* in mice resulted in infertility, prostatic hyperplasia, and dysplastic foci and increased pAKT in vivo [39]. Therefore, we examined β-catenin and pAKT protein expression in scrambled control or anti-miR-186-5p transfected PC-3 and MDA-PCa-2b cells relative to appropriate scramble controls (Fig. 5e-h). Inhibition of miR-186-5p significantly reduced β-catenin protein in MDA-PCa-2b and PC-3 cells (Fig. 5e, g). Similarly, inhibition of miR-186-5p decreased pAKT levels in PC-3 whole cell lysates, but not in MDA-PCa-2b cells (Fig. 5f, h). No change in total AKT was detected in PC-3 and MDA-PCa-2b cells transfected with anti-miR-186-5p (Fig. 5f, h). Collectively, the aforementioned data support a pathway by which miR-186-5p inhibition upregulates AKAP12 and decreases pAKT and β-catenin in some metastatic PCa cells, as modeled in Fig. 6.

## Discussion

Altered miRNA profiles contribute to dysregulation of gene expression involved in the pathogenesis of metastatic PCa [40]. In the current study, we identified upregulation of miR-186-5p in the serum from PCa patients (tumor stage I, III and IV) and metastatic PCa cell lines (LNCaP, MDA-PCa-2b, PC-3) relative to their respective controls. We also demonstrated inhibition of miR-186-5p reduced PCa cell proliferation, anchorage-independent cell growth, colony formation, and invasion of metastatic PC-3 and/or MDA-PCa-2b cells. Collectively, these findings suggest miR-186-5p plays an oncogenic role in PCa. Indeed, ectopic miR-186-5p expression enhanced anchorage independent growth of LNCaP cells but not PC-3 or MDA-PCa-2b cells.

Whether miR-186 plays a tumor suppressor or oncogenic role in cancer progression may depend on the cancer type and stage of the disease [13, 14, 17, 20–25, 27, 29–32, 41]. Some reports suggest miR-186 plays a tumor suppressor role and targets oncogenic-related genes in NSCLC [23, 24], ovarian [21], oral squamous [42], bladder [43], pancreatic [17], multiple myeloma [44], cervical [45], esophageal [46], gastric [47], hepatocellular [48], renal [49], and glioblastoma multiforme [50] cancer cells. In contrast, other reports implicate an oncogenic role for miR-186. For example, in agreement with our data in PCa cells, inhibition of miR-186 decreased cell proliferation and invasion of pancreatic [17], bladder [20], and colon [51] cancer cell lines. It is important to note these studies do not clarify whether they evaluated miR-186-3p or miR-186-5p. However, the current study demonstrated the miR-186-5p form may have an oncogenic role in prostate cancer.

There are limited reports on the role of miR-186 in PCa [28–32, 41]. Commensurate with our serum and in vitro findings, Ambs and colleagues (2008) observed higher miR-186-5p expression in laser micro-dissected tumor tissue from PCa patients diagnosed with extra-prostatic disease relative to patients with no extra-prostatic disease among European (*n* = 30) and African American (*n* = 30) men [28]. In contrast, other studies reported miR-186 was down-regulated in non-microdissected PCa tissue [30, 31] and PCa cell lines, i.e.*,* M12, P69, PC-3, Tsu-Pr1, LNCaP, 22Rv1, DU145 [30–32]. Collectively, these studies suggest a tumor suppressor role for miR-186 in PCa. However, these authors do not distinguish whether the miR-186 precursor, miR-186-5p or miR-186-3p were responsible for apparent cell effects of miR-186 overexpression in PCa cell lines or tissue. Our report suggests miR-186-5p may have an oncogenic role due to its up-regulation in the serum of PCa patients and tumor cell lines. The discrepancies between our findings and other miR-186 reports may also be attributed to the following: (1) tumor tissue processing or storage methods; (2) selection of “normal prostate” tissue using micro-dissected versus non-microdissected tissue; (3) types of “control” cell lines used for comparison purposes; (4) degree of specificity of primers used for quantitation of miR-186-3p or miR-186-5p; (5) selection of normalizer for miRNA quantification; and (6) methods of miRNA isolation and detection. For the detection and semi-quantitation of miR-186-5p in the current study, we used normal epithelial cell lines (e.g., RWPE1) for comparison purposes, the miRVana miRNA isolation kit, primers specific for miR-186-5p, and U44 for normalization. In the current study, the U44 levels did not vary among normal epithelial and the prostate cancer cell lines (data not shown). The studies that indicated an tumor suppressor role for miR-186 used: (1) primary culture prostate epithelial cells for comparison purposes with no further details about these cell models; (2) snoRNAs U24, or U6 as controls to normalize miRNA levels; (3) varying kits for miRNA isolation (i.e., Trizol, Qiagen miRNeasy mini kit, E.Z.N.A.® miRNA kit); and (4) non-specific rather than specific miRNA primers [30–32]. To our knowledge, the current study is the first to demonstrate the up-regulation of miR-186-5p in serum from PCa patients and in metastatic PCa cell lines. For the serum based studies, we used the miRVana isolation kit and an exogenous miRNA control, namely cel-miR-39 (i.e., *C. Elegans* 39 – a nonhuman miRNA).

We identified miR-186-5p targets using *in silico* tools followed by functional assays. Among the established miR-186-5p targets, we selected AKAP12 for further evaluation due to its central role in cell proliferation, colony formation, cell invasion and epithelial mesenchymal transition and tumor growth [39, 52–56]. A previous study demonstrated AKAP12 is a bona fide direct target of miR-186-5p using 3’-UTR-luciferase reporter assay [57]. Down-regulation of AKAP12 suppresses cell proliferation, survival, motility, migration, anchorage independent growth, angiogenesis and invasion in several cancers (reviewed in [52]).

Notably, several cancer phenotypes (i.e., cell proliferation, colony formation, invasion) were attenuated upon miR-186-5p inhibition in metastatic PCa cell lines in the current study. This reduction in aggressive PCa behavior may partially correspond with upregulation of AKAP12. Ectopic expression of AKAP12 is associated with a decrease in cell invasion and anchorage independent growth of mouse PCa cells [54, 58]. Furthermore, knockout of AKAP12 in mice resulted in prostatic hyperplasia and dysplastic foci [39], supporting a role of AKAP12 as a tumor suppressor gene.

AKAP12 knockout mice also showed increased expression of pAKT in prostate tissue [39]. pAKT signaling inhibits Glycogen Synthase Kinase-3 (GSK3) activity, which leads to β-catenin nuclear accumulation [38]. Loss of tumor suppressor PTEN, which is common in PCa, leads to activation of PI3K and AKT signaling and subsequent β-catenin phosphorylation [59]. Phosphorylation of β-catenin increases its stability and nuclear localization, which leads to its association with T-cell specific transcription factor/lymphoid enhancer-binding factor 1 family proteins (TCF/LEF-1) in the nucleus. The TCF/LEF-1-β-catenin complex recruits coactivators such as B-cell lymphoma 9 protein (Bcl-9) and cAMP response element binding protein (CREB)-binding protein (CBP) [60], resulting in an increase in the transcription of genes involved in tumorigenesis, angiogenesis, extracellular matrix, and cell cycle progression, including *MYC, MMP7*, *VEGF*, and *CCND1* [61, 62].

In addition, β-catenin is an integral signaling protein for epithelial mesenchymal transition (EMT) and the transcription of genes in the canonical Wnt signaling pathway [38]. Here, we observed inhibition of miR-186-5p resulted in a decrease in β-catenin protein in PC-3 and MDA-PCa-2b cells. We speculate the repression of the aggressive PCa phenotype observed in miR-186 inhibited PCa cells is partially attributed to AKAP12 mediated down-regulation of pAKT, which in turn, may down-regulate β-catenin (modeled in Fig. 6). Several reports demonstrate pAKT phosphorylates and stabilizes β-catenin and phosphorylated β-catenin activates AKT expression [31, 63]. Consequently, we propose an increase in miR-186-5p expression stimulates AKT signaling via decreasing AKAP12, leading to increased nuclear β-catenin, an EMT mediator. This proposed mechanism may explain the influence of miR-186-5p on cell invasion and anchorage-independent growth in metastatic PCa cell models in the current study. Future studies will validate whether miR-186-5p inhibition down-regulates AKT signaling, allowing GSK3 to promote β-catenin degradation.

We evaluated the strengths, limitations and future directions of the current study. In a pilot study, our lab was the first to demonstrate the up-regulation of miR-18-5p in the serum of patients diagnosed with non-metastic and metastatic prostate cancer when compared to those without prostate cancer. Although the controls on average had elevated PSA, they were designated as prostate cancer-free at the time of the serum collection, following a biopsy. We cannot exclude the possibility that the controls had benign prostatic hyperplasia. If we assume the controls in the current study had BPH, we would anticipate an even higher fold increase serum-based miR-186-5p levels from prostate cancer patients relative to levels among non-BPH controls. Future studies will confirm whether miRNA-186-5p detected in serum and matched micro-dissected prostate tumor specimen correspond with high tumor grade/stage, metastatic disease, higher risk of biochemical/disease recurrence, or hormone refractory status within large and ethnically diverse patient sub-groups.

Although miR-186-5p was also up-regulated in non-metastatic and metastatic prostate cancer cell lines, miR-186-5p levels did not vary by androgen receptor status. Interestingly, over-expression of miR-186-5p in PC-3 cell lines did not result in an increase in aggressive prostate cancer cellular behavior. Since the PC-3 cells have the highest expression of miR-186-5p, we believe overexpression of miR-186-5p in PC-3 cells had no further biological effect in these cells. The high baseline levels of miR-186-5p in the PC3 cells may have saturated any biological effects (i.e., cell proliferation, colony formation and cell invasion) and prevented our capacity to detect further aggressive phenotypes in these already transformed cells. However, additional pre-clinical studies are needed to assess whether miR-186-5p overexpression and inhibition alter aggressive tumor behavior in other metastatic PCa cell models (e.g., TSU-Pr1, MDA PCa-2a, VCaP, DuCaP) as well as tumorigenesis in animal models in the presence and absence of androgen stimulation.

To our knowledge, this is the first study to demonstrate AKAP12 as a miR-186-5p target in metastatic PCa cell lines (PC-3, MDA-PCa-2b). Importantly, inhibition of miR-186-5p suppressed three metastatic PCa cell hallmarks, namely proliferation, invasion, and colony growth. We speculate the reduction in cell proliferation, invasion, and anchorage independent growth with anti-miR-186-5p may be attributed, at least in part, to AKAP12’s role in pAkt mediated suppression of β-catenin. Following inhibition/overexpression of miR-186-5p, we demonstrated an up-regulation of tumor suppressor AKAP12 as well as a downregulation pAkT and β-catenin in vitro in total cell lysates of PC3 cells. Since increased β-catenin is apparent in many cancers, including prostate cancer [4, 5, 7], future studies will focus on the evaluation of β-catenin levels in both the cytoplasm and nucleus, following inhibition/overexpression of miR-186-5p using in vitro cell models. Apparently, the translocation of β-catenin from the cytoplasm to the nucleus is where it mediates the transcription of target genes related to EMT and metastasis. Since B-catenin mediates its effects on EMT markers.

In our microarray analysis, we observed previously published miR-186 validated targets (*AKAP12, ROCK1, PPM1B*, and *PTTG1*) were downregulated as anticipated in RWPE1 stably transfected with miR-186-5p and/or up-regulated in PC-3 cells stably transfected with anti-miR-186. Although hundreds of potential miR-186-5p targets were identified in the miR-186-5p depleted PC-3 cells, we focused on AKAP12, a validated target involved in prostate cancer. We also focused on AKAP12 because it plays a role in many of cell behaviors (e.g., cell proliferation, colony formation, cell invasion, cell motility, EMT, metastasis, cell invasion, cell cycle arrest, and cell death) that were also modified in our miR-186-5p suppressed metastatic cell models. AKAP12 appears to mediate its effects on cell invasion, presumably through the p-Akt/β-catenin the pathway. In future studies, our lab will validate both common and unique targets revealed in the top 30 targets in anti-miR-186-5p transfected PC-3 and miR-186-5p overexpressing prostate cancer cell models cells.

## Conclusions

This is the first report that miR-186-5p is upregulated in PCa patient serum and cell lines. We demonstrated inhibition of miR-186-5p inhibited anchorage-independent cell growth and invasion and reduced pAKT and β-catenin levels, while increasing tumor suppressor AKAP12. Overall, our data suggest miR-186-5p may function in an oncogenic capacity and serve as a potential prognostic tool and therapeutic target in PCa. Lastly, future studies will elucidate how the miR-186-5p-AKAP12 axis and other miR-186-5p targets play a role in prostate cancer using in vitro and in vivo models.

## Additional files

Additional file 1 **Table S1.** De-identified demographic and clinico-pathological data. Clinical data and serum from 15 men diagnosed with prostate cancer and five disease-free individuals were obtained from the BioServe Biotechnologies Biorepository (Beltsville, MD). Subjects were self-identified as European American males. There were no significant differences in median age between cases and controls (*p* = 0.726). Among men diagnosed with prostate cancer, 60% were diagnosed with adenocarcinoma, 66.7% were smokers, and 73.3% received two or more therapies. Relative to controls, cases had higher median serum PSA level (ng/ml) (*p* = 0.048) and BMI (*p* = 0.225) values. (DOCX 63 kb)

Additional file 2 **Table S2.** Differentially expressed human miRNAs in serum from PCa patients. Relative to disease-free individuals, human miRNAs were down-regulated (fold change ≤ − 1.5) and up-regulated (fold change ≥1.5) in PCa patients when compared to disease-free individuals based on Taqman Human MicroRNA Array data (*p*-value ≤0.05). Global normalization of miRNA profiles identified 26 differentially expressed human miRNAs (9 down-regulated and 17 up-regulated) in the serum from patients diagnosed with tumor stage I (*n* = 5), III (*n* = 5) and IV (*n* = 5) relative to disease-free individuals (*n* = 5). miRNAs (miRs-106b-5p, − 186-5p, −302b-3p, − 342-3p, −520e, − 885-5p), highlighted in gray, represent targets that survived multiple hypothesis testing (FDR *p*-value ≤0.05). (DOCX 19 kb)

Additional file 3 **Figure S1.** Transient and stable miR-186-5p inhibition and overexpression in prostate cancer and normal epithelial cells. miR-186-5p expression was measured following 24–48 h transient post-transfection and stable transfection using qRT-PCR. A) Following transient transfection of cell models with miR-186-5p inhibitor, miR-186-5p was reduced by 33–73% in PC-3 (*p* = 0.0002), MDA-PCA-2b (*p* = 0.0061) and LNCaP (*p* = 0.0381) cells. B) Ectopic expression of miR-186-5p in PC-3 (*p* = 0.0002), MDA-PCA-2b (*p* = 0.0061) and LNCaP (*p* = 0.0381) cells transiently transfected with miR-186-5p mimic. C) MiR-186-5p expression was reduced by 30% in PC-3 cells stably transfected with pcDNA-DEST-47-anti-miR-186 compared to pcDNA-DEST-47 (empty vector) (*p* = 0.0022). MiR-186-5p was up-regulated by 2.55-fold in RWPE1 cells stably transfected with pcDNA-DEST-47-miR-186 mimic construct compared to the empty vector control (*p* = 0.0019). Data was quantitated from three independent experiments and are represented as mean fold change ± S.D. (**p-value < 0.005, ***p-value < 0.007). (TIFF 1824 kb)

Additional file 4 **Table S3.** Aberrant gene expression in miR-186-5p inhibited PC-3 and miR-186-5p overexpressing RWPE1 cells. Microarray gene list was restricted to genes down-regulated in RWPE1 (fold change ≤ − 1.2) and up-regulated in PC-3 cells (fold change ≥1.2). Analysis revealed a down-regulation of 493 transcripts in RWPE1 cells and up-regulation of 547 transcripts in PC-3 cells. Genes in bold represent previously validated miR-186-5p targets. Moreover, genes were highlighted gray if modified in both RWPE1 with ectopic expression of miR-186-5p and miR-186-5p inhibited PC-3 cells. (DOCX 81 kb)

Additional file 5 **Figure S2.** Up-regulation of AKAP12 in PC-3 cells and pAKT in HEK 293 T cells. A) Total RNA was collected from PC-3 cells transfected with miR-186-5p inhibitor and scramble control 72 h post-transfection. AKAP12 transcript expression was increased by 1.7-fold in transient miR-186-5p inhibited PC-3 cells relative to negative controls (*p* = 0.0597). B) Protein lysate (35 μg) was collected from HEK 293 T cells transfected with miR-186-5p mimic and scramble control 72 h post-transfection. pAKT expression was enhanced by 1.67-fold increase via miR-186-5p overexpression in HEK 293 T cells (*p* = 0.196). Data was quantitated from at least 2–3 independent experiments and are represented as mean ± S.D. (TIFF 457 kb)

## Abbreviations

AKAP12: A-kinase anchor protein 12 = AKAP12
BMI: Body mass index = BMI
cDNA: Complementary DNA
CTBNB1: β-catenin
EMT: Epithelial mesenchymal transition
miRs or miRNAs: MicroRNAs
pAKT: Phosphorylated-AKT serine/threonine-protein kinase
PCa: Prostate cancer
PKA: Protein kinase A
PKC: Protein kinase C
PSA: Prostate serum antigen
qRT-PCR: Quantitative Real-time PCR
S.D.: Standard deviation
