# Supplementary material for: Micro-RNA-186-5p inhibition attenuates proliferation, anchorage independent growth and invasion in metastatic prostate cancer cells
Source: BMC Cancer. 2018 Apr 13;18:421. doi: 10.1186/s12885-018-4258-0 (PMC5899400; doi:10.1186/s12885-018-4258-0)
Supplement: Supplementary file 1 — Table S1. De-identified demographic and clinico-pathological data. Clinical data and serum from 15 men diagnosed with prostate cancer and five disease-free individuals were obtained from the BioServe Biotechnologies Biorepository (Beltsville, MD). Subjects were self-identified as European American males. There were no significant differences in median age between cases and controls (p = 0.726). Among men diagnosed with prostate cancer, 60% were diagnosed with adenocarcinoma, 66.7% were smokers, and 73.3% received two or more therapies. Relative to controls, cases had higher median serum PSA level (ng/ml) (p = 0.048) and BMI (p = 0.225) values. (DOCX 63 kb) [file 12885_2018_4258_MOESM1_ESM.docx]

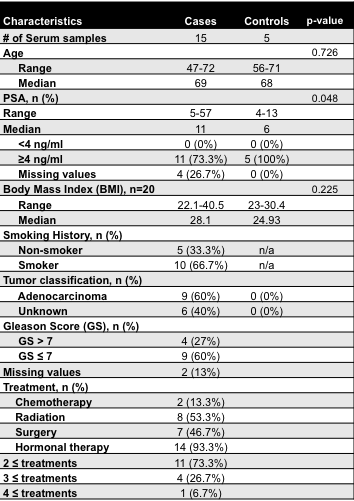


**Additional File 1: Table S1. De-identified demographic and clinico-pathological data.** Clinical data and serum from fifteen men diagnosed with prostate cancer and five disease-free individuals were obtained from the BioServe Biotechnologies Biorepository (Beltsville, MD). Subjects were self-identified as European American males. There were no significant differences in median age between cases and controls (p = 0.726). Among men diagnosed with prostate cancer, 60% were diagnosed with adenocarcinoma, 66.7% were smokers, and 73.3% received two or more therapies. Relative to controls, cases had higher median serum PSA level (ng/ml) (p = 0.048) and BMI (p = 0.225) values.
