# Supplementary material for: Micro-RNA-186-5p inhibition attenuates proliferation, anchorage independent growth and invasion in metastatic prostate cancer cells
Source: BMC Cancer. 2018 Apr 13;18:421. doi: 10.1186/s12885-018-4258-0 (PMC5899400; doi:10.1186/s12885-018-4258-0)
Supplement: Supplementary file 2 — Table S2. Differentially expressed human miRNAs in serum from PCa patients. Relative to disease-free individuals, human miRNAs were down-regulated (fold change ≤ − 1.5) and up-regulated (fold change ≥1.5) in PCa patients when compared to disease-free individuals based on Taqman Human MicroRNA Array data (p-value ≤0.05). Global normalization of miRNA profiles identified 26 differentially expressed human miRNAs (9 down-regulated and 17 up-regulated) in the serum from patients diagnosed with tumor stage I (n = 5), III (n = 5) and IV (n = 5) relative to disease-free individuals (n = 5). miRNAs (miRs-106b-5p, − 186-5p, −302b-3p, − 342-3p, −520e, − 885-5p), highlighted in gray, represent targets that survived multiple hypothesis testing (FDR p-value ≤0.05). (DOCX 19 kb) [file 12885_2018_4258_MOESM2_ESM.docx]

|  | **Stage I** | | **Stage III** | | **Stage IV** | |
| --- | --- | --- | --- | --- | --- | --- |
| **miRNA/Assay #** | **p-value** | **FDR p-value** | **p-value** | **FDR p-value** | **p-value** | **FDR p-value** |
| hsa-let-7b-002619 |  |  | 0.034 | 0.242 |  |  |
| hsa-miR-106b-000442 | 0.001 | 0.038 | 0.001 | 0.016 | 0.001 | 0.044 |
| hsa-miR-122-002245 | 0.003 | 0.065 | 0.007 | 0.099 | 0.002 | 0.053 |
| hsa-miR-133a-002246 |  |  | 0.026 | 0.199 | 0.041 | 0.412 |
| hsa-miR-142-3p-000464 | 0.022 | 0.167 |  |  |  |  |
| hsa-miR-145-002278 | 0.027 | 0.186 | 0.021 | 0.187 | 0.018 | 0.311 |
| hsa-miR-150-000473 |  |  |  |  | 0.009 | 0.241 |
| hsa-miR-185-002271 | 0.015 | 0.147 |  |  |  |  |
| hsa-miR-186-002285 | 0.012 | 0.147 | 1.02E-04 | 0.005 |  |  |
| hsa-miR-18a-002422 | 0.019 | 0.167 |  |  |  |  |
| hsa-miR-191-002299 | 0.046 | 0.242 | 0.012 | 0.144 |  |  |
| hsa-miR-197-000497 | 0.043 | 0.240 |  |  | 0.047 | 0.412 |
| hsa-miR-21-000397 | 0.003 | 0.065 | 0.004 | 0.062 | 0.015 | 0.311 |
| hsa-miR-296-000527 |  |  |  |  | 0.022 | 0.328 |
| hsa-miR-302b-000531 | 1.12E-04 | 0.012 | 7.69E-06 | 0.001 |  |  |
| hsa-miR-320-002277 |  |  |  |  | 0.040 | 0.412 |
| hsa-miR-342-3p-002260 | 5.00E-04 | 0.026 | 0.001 | 0.018 | 2.63E-04 | 0.028 |
| hsa-miR-374-000563 | 0.021 | 0.167 |  |  |  |  |
| hsa-miR-454-002323 |  |  | 0.026 | 0.199 |  |  |
| hsa-miR-484-001821 |  |  | 0.019 | 0.187 |  |  |
| hsa-miR-518d-001159 | 0.028 | 0.186 |  |  |  |  |
| hsa-miR-520e-001119 | 0.013 | 0.147 | 0.001 | 0.018 |  |  |
| hsa-miR-744-002324 |  |  |  |  | 0.044 | 0.412 |
| hsa-miR-885-5p-002296 |  |  | 3.14E-04 | 0.011 |  |  |
| hsa-miR-92a-000431 | 0.013 | 0.147 |  |  |  |  |
| hsa-miR-99b-000436 | 0.033 | 0.205 |  |  |  |  |

**Additional File 2: Table S2.** **Differentially expressed human miRNAs in serum from PCa patients.** Relative to disease-free individuals, human miRNAs were down-regulated (fold change ≤ -1.5) and up-regulated (fold change ≥ 1.5) in PCa patients when compared to disease-free individuals based on Taqman Human MicroRNA Array data (p-value ≤ 0.05). Global normalization of miRNA profiles identified 26 differentially expressed human miRNAs (9 down-regulated and 17 up-regulated) in the serum from patients diagnosed with tumor stage I (n = 5), III (n = 5) and IV (n = 5) relative to disease-free individuals (n = 5). miRNAs (miRs-106b-5p, -186-5p, -302b-3p, -342-3p, -520e, -885-5p), highlighted in gray, represent targets that survived multiple hypothesis testing (FDR p-value ≤ 0.05).
