# Supplementary figures and images for: Micro-RNA-186-5p inhibition attenuates proliferation, anchorage independent growth and invasion in metastatic prostate cancer cells
Source: BMC Cancer. 2018 Apr 13;18:421. doi: 10.1186/s12885-018-4258-0 (PMC5899400; doi:10.1186/s12885-018-4258-0)

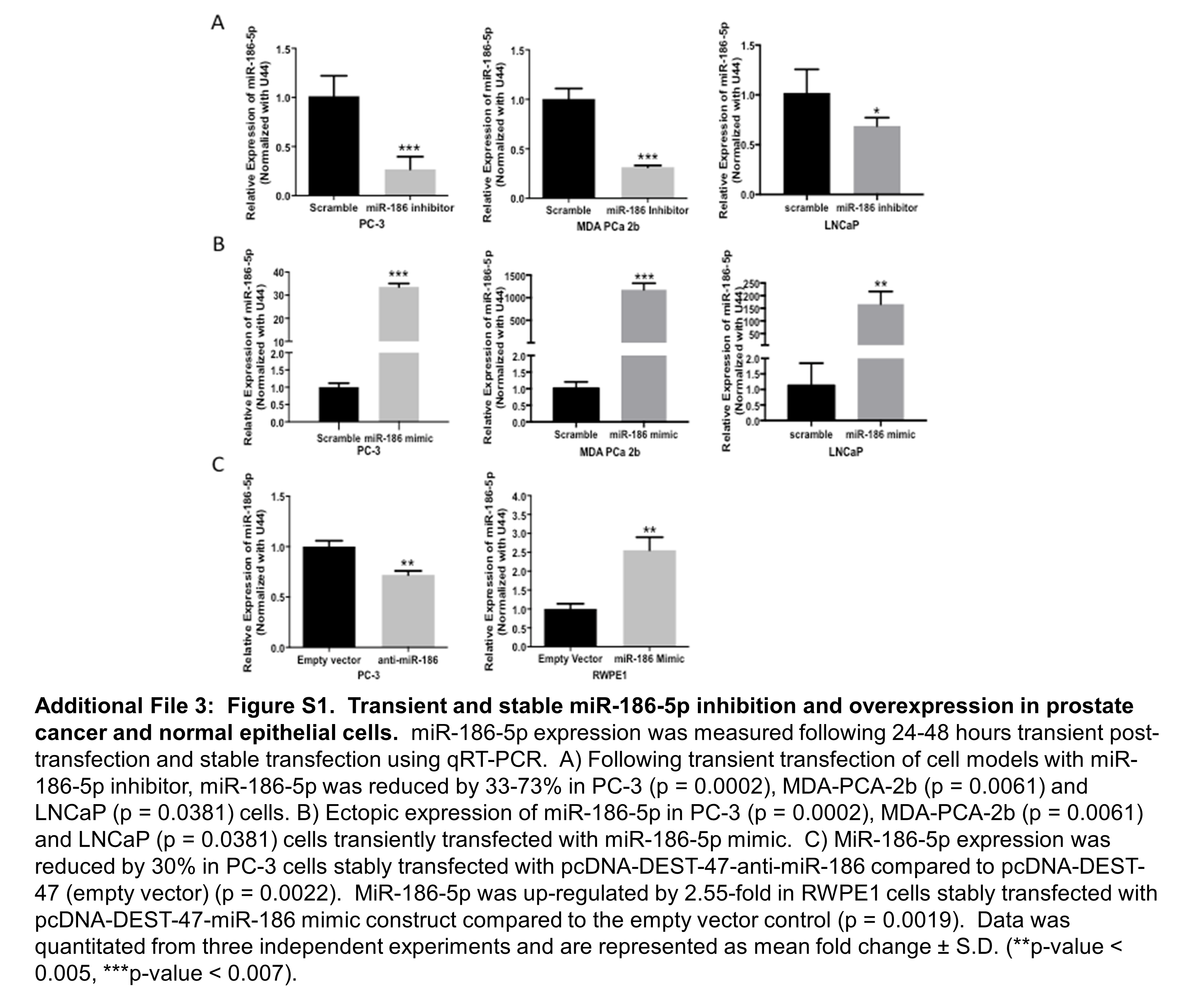

Supplement: Supplementary file 3 — Figure S1. Transient and stable miR-186-5p inhibition and overexpression in prostate cancer and normal epithelial cells. miR-186-5p expression was measured following 24–48 h transient post-transfection and stable transfection using qRT-PCR. A) Following transient transfection of cell models with miR-186-5p inhibitor, miR-186-5p was reduced by 33–73% in PC-3 (p = 0.0002), MDA-PCA-2b (p = 0.0061) and LNCaP (p = 0.0381) cells. B) Ectopic expression of miR-186-5p in PC-3 (p = 0.0002), MDA-PCA-2b (p = 0.0061) and LNCaP (p = 0.0381) cells transiently transfected with miR-186-5p mimic. C) MiR-186-5p expression was reduced by 30% in PC-3 cells stably transfected with pcDNA-DEST-47-anti-miR-186 compared to pcDNA-DEST-47 (empty vector) (p = 0.0022). MiR-186-5p was up-regulated by 2.55-fold in RWPE1 cells stably transfected with pcDNA-DEST-47-miR-186 mimic construct compared to the empty vector control (p = 0.0019). Data was quantitated from three independent experiments and are represented as mean fold change ± S.D. (**p-value < 0.005, ***p-value < 0.007). (TIFF 1824 kb) [file 12885_2018_4258_MOESM3_ESM.tif]

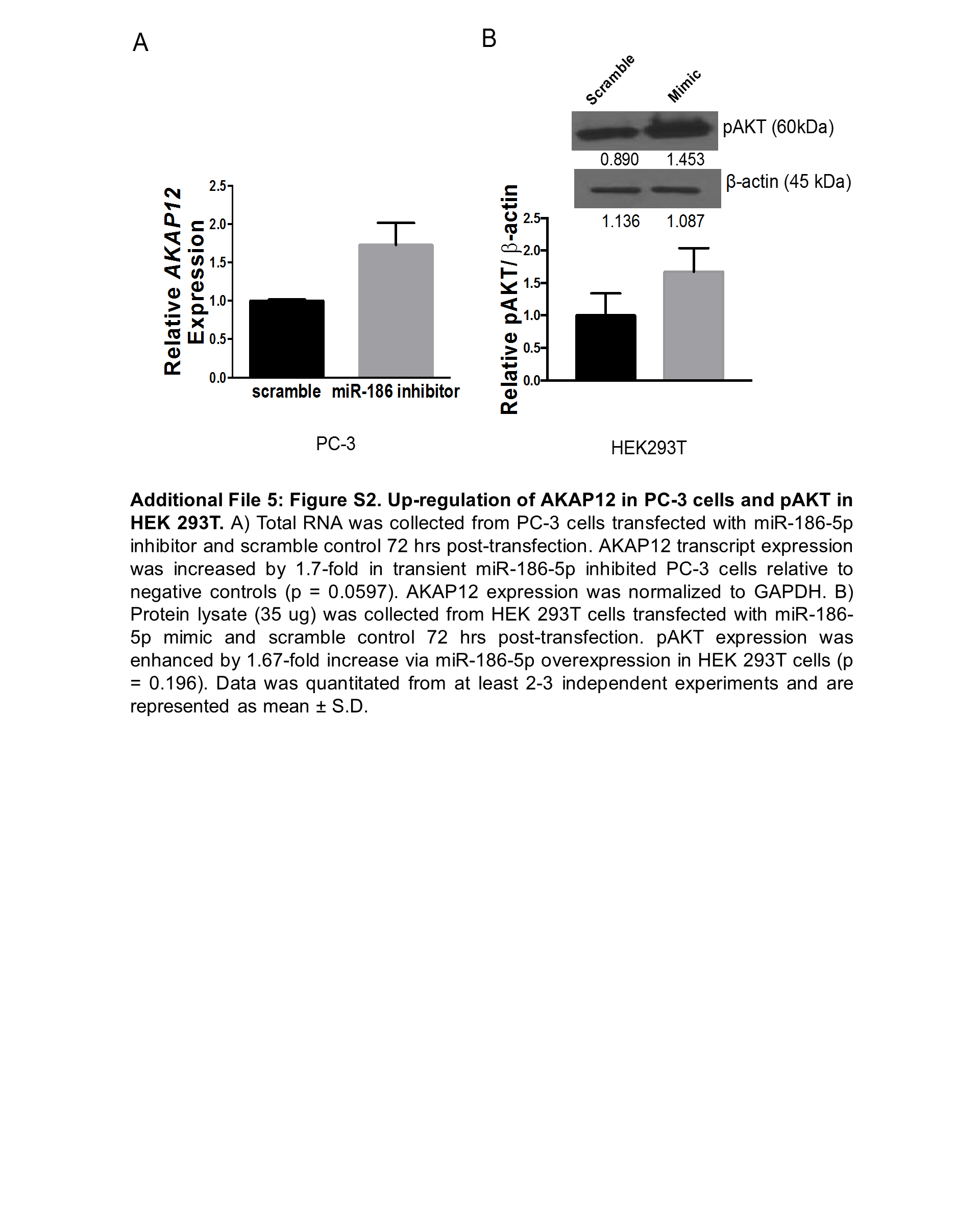

Supplement: Supplementary file 5 — Figure S2. Up-regulation of AKAP12 in PC-3 cells and pAKT in HEK 293 T cells. A) Total RNA was collected from PC-3 cells transfected with miR-186-5p inhibitor and scramble control 72 h post-transfection. AKAP12 transcript expression was increased by 1.7-fold in transient miR-186-5p inhibited PC-3 cells relative to negative controls (p = 0.0597). B) Protein lysate (35 μg) was collected from HEK 293 T cells transfected with miR-186-5p mimic and scramble control 72 h post-transfection. pAKT expression was enhanced by 1.67-fold increase via miR-186-5p overexpression in HEK 293 T cells (p = 0.196). Data was quantitated from at least 2–3 independent experiments and are represented as mean ± S.D. (TIFF 457 kb) [file 12885_2018_4258_MOESM5_ESM.tif]
