# Supplementary material for: Micro-RNA-186-5p inhibition attenuates proliferation, anchorage independent growth and invasion in metastatic prostate cancer cells
Source: BMC Cancer. 2018 Apr 13;18:421. doi: 10.1186/s12885-018-4258-0 (PMC5899400; doi:10.1186/s12885-018-4258-0)
Supplement: Supplementary file 4 — Table S3. Aberrant gene expression in miR-186-5p inhibited PC-3 and miR-186-5p overexpressing RWPE1 cells. Microarray gene list was restricted to genes down-regulated in RWPE1 (fold change ≤ − 1.2) and up-regulated in PC-3 cells (fold change ≥1.2). Analysis revealed a down-regulation of 493 transcripts in RWPE1 cells and up-regulation of 547 transcripts in PC-3 cells. Genes in bold represent previously validated miR-186-5p targets. Moreover, genes were highlighted gray if modified in both RWPE1 with ectopic expression of miR-186-5p and miR-186-5p inhibited PC-3 cells. (DOCX 81 kb) [file 12885_2018_4258_MOESM4_ESM.docx]

| **Gene (PC-3)** | **Fold change** | **FDR p-value** | **Gene (RWPE1)** | **Fold change** | **FDR p-value** |
| --- | --- | --- | --- | --- | --- |
| PMEPA1 | 6.255 | 8.50219E-12 | EHF | -6.261 | 2.15476E-10 |
| FN1 | 5.820 | 5.69038E-10 | ZNF711 | -4.141 | 1.78879E-09 |
| TSC22D3 | 4.701 | 1.20663E-06 | GPC6 | -3.327 | 1.39841E-06 |
| EFEMP1 | 4.334 | 3.61878E-07 | FOXG1 | -2.929 | 0.000587093 |
| EGR1 | 4.130 | 8.19665E-07 | CPE | -2.769 | 4.23842E-05 |
| TUBE1 | 3.931 | 1.90071E-08 | CLCA2 | -2.613 | 0.000051171 |
| JAG1 | 3.229 | 4.83412E-06 | CCL20 | -2.581 | 6.13709E-05 |
| ZNF674 | 2.983 | 8.39119E-07 | SPX | -2.496 | 4.34943E-07 |
| SLITRK6 | 2.911 | 6.95917E-06 | ZFP42 | -2.431 | 3.0742E-06 |
| PSAT1 | 2.587 | 3.20654E-09 | **AKAP12** | -2.376 | 0.000243387 |
| KCNT2 | 2.489 | 1.49162E-06 | ALDH1A2 | -2.321 | 3.93333E-05 |
| VEGFA | 2.489 | 4.31602E-11 | HECTD2 | -2.319 | 0.000018492 |
| CEBPG | 2.472 | 4.43309E-11 | MCTP1 | -2.239 | 1.89756E-07 |
| KLF9 | 2.426 | 7.74733E-06 | APOLD1 | -2.228 | 0.000016442 |
| WNT5A | 2.426 | 2.56651E-07 | HMGN5 | -2.183 | 0.000131712 |
| TRIM36 | 2.403 | 7.29682E-07 | TPRG1 | -2.182 | 7.81032E-06 |
| SLC22A15 | 2.399 | 3.03846E-05 | GPR19 | -2.172 | 3.87728E-06 |
| CSGALNACT1 | 2.374 | 2.85369E-07 | BCL11A | -2.145 | 6.80441E-05 |
| RHOB | 2.328 | 1.55029E-05 | GJA3 | -2.036 | 1.67961E-05 |
| SAT1 | 2.291 | 5.84464E-10 | BCL2L11 | -2.030 | 4.64641E-05 |
| MAML3 | 2.288 | 0.000130184 | TLR2 | -1.994 | 4.82555E-05 |
| ERRFI1 | 2.200 | 1.12777E-10 | OLR1 | -1.984 | 0.000261123 |
| KDM7A | 2.193 | 2.32714E-06 | RNF125 | -1.976 | 5.22618E-05 |
| ZNF558 | 2.179 | 0.000232804 | SLC7A2 | -1.959 | 9.12173E-05 |
| CD55 | 2.121 | 6.2058E-07 | IL15 | -1.959 | 0.0138083 |
| APOBEC3F | 2.112 | 0.000469815 | BCL11B | -1.925 | 0.000020977 |
| PLEKHG1 | 2.081 | 2.30428E-05 | GSTM3 | -1.896 | 3.33722E-08 |
| MTHFD2 | 2.048 | 3.7309E-12 | PPP2R2C | -1.892 | 0.00076474 |
| BCL6 | 2.039 | 5.70622E-05 | FBN2 | -1.866 | 0.0190539 |
| C20orf197 | 2.011 | 0.000166965 | PCDH7 | -1.864 | 0.000575647 |
| SGK1 | 1.996 | 1.51012E-09 | BTNL9 | -1.849 | 1.76586E-05 |
| ANK2 | 1.981 | 8.54983E-05 | PCGF5 | -1.830 | 5.66145E-06 |
| LMO4 | 1.975 | 5.79935E-07 | PABPC4L | -1.829 | 6.97385E-06 |
| CALB1 | 1.973 | 3.09243E-05 | EGR1 | -1.823 | 0.00435888 |
| RNF125 | 1.925 | 4.21354E-05 | MTSS1 | -1.812 | 0.000830112 |
| COL4A2 | 1.889 | 0.000159881 | RPL31 | -1.807 | 0.000211004 |
| HDX | 1.889 | 0.00145928 | STAT4 | -1.802 | 0.000394484 |
| JUN | 1.880 | 5.07925E-05 | GOLT1B | -1.792 | 1.18469E-06 |
| FKTN | 1.875 | 1.10802E-06 | SCD5 | -1.787 | 1.33662E-06 |
| USP53 | 1.875 | 1.05228E-07 | AUTS2 | -1.783 | 0.00177085 |
| TFAP2A | 1.875 | 7.61731E-05 | SLC6A15 | -1.773 | 7.19014E-06 |
| STAG3L4 | 1.858 | 8.55957E-08 | PLEKHA5 | -1.771 | 3.81374E-07 |
| CSTA | 1.852 | 0.00018635 | LBR | -1.762 | 1.2621E-07 |
| NFKBIZ | 1.836 | 9.80932E-07 | MXI1 | -1.756 | 1.65509E-05 |
| SEPSECS | 1.825 | 1.99296E-05 | HHEX | -1.749 | 1.75521E-07 |
| ZNF528 | 1.824 | 0.00325158 | KLHL24 | -1.747 | 0.022777 |
| TSPAN31 | 1.792 | 1.30804E-05 | MLF1 | -1.744 | 6.92344E-06 |
| RND3 | 1.791 | 7.91426E-06 | PPFIBP1 | -1.734 | 0.000875545 |
| MME | 1.785 | 0.000928321 | FOXJ2 | -1.719 | 4.52903E-05 |
| KLHL24 | 1.778 | 0.01464 | CD24 | -1.714 | 6.89321E-08 |
| **Gene (PC-3)** | **Fold change** | **FDR p-value** | **Gene (RWPE1)** | **Fold change** | **FDR p-value** |
| C5orf28 | 1.767 | 2.63717E-06 | SLC47A1 | -1.699 | 0.000043448 |
| ABCA1 | 1.766 | 2.63717E-06 | ADIPOR2 | -1.688 | 9.42056E-09 |
| MICAL2 | 1.758 | 5.54121E-06 | PIK3AP1 | -1.672 | 0.000240404 |
| PMS2CL | 1.754 | 3.36844E-06 | HCP5 | -1.665 | 0.000429795 |
| POLI | 1.752 | 0.0069011 | IL17RD | -1.664 | 0.00060462 |
| YPEL2 | 1.746 | 0.0221395 | NIPAL4 | -1.663 | 0.000783609 |
| RAP2B | 1.744 | 0.00127713 | ZNF382 | -1.660 | 0.000677792 |
| NR1D2 | 1.743 | 0.00291668 | NEK2 | -1.658 | 9.75577E-05 |
| CXorf23 | 1.742 | 0.00298402 | MANSC1 | -1.651 | 3.01405E-06 |
| PPP4R4 | 1.739 | 0.000892712 | NRIP3 | -1.644 | 0.000272348 |
| IRF2BP2 | 1.739 | 1.71221E-07 | STK17B | -1.644 | 4.63227E-07 |
| TSEN15 | 1.736 | 1.39297E-07 | KIAA0895 | -1.627 | 0.00320574 |
| SKIL | 1.732 | 0.00328462 | ZBTB41 | -1.625 | 5.17397E-05 |
| EML2 | 1.732 | 1.35962E-05 | KCNMB4 | -1.608 | 0.00301134 |
| PARP11 | 1.729 | 1.50634E-05 | SYCP2 | -1.608 | 0.00152086 |
| C4orf32 | 1.717 | 7.31696E-07 | FAM72A | -1.595 | 6.36124E-05 |
| NRP1 | 1.711 | 1.26178E-06 | FAM72B | -1.595 | 6.36124E-05 |
| COL4A1 | 1.701 | 1.14459E-06 | KLF9 | -1.594 | 0.00400261 |
| DLC1 | 1.697 | 0.00167147 | FAM117B | -1.594 | 6.08913E-05 |
| SLC7A8 | 1.696 | 0.0122188 | TMEM71 | -1.591 | 0.000038077 |
| CNTNAP3 | 1.694 | 5.84032E-06 | KLHL28 | -1.589 | 0.00119398 |
| ALDH1L2 | 1.689 | 0.00115116 | IGSF3 | -1.589 | 0.0092947 |
| RSL24D1 | 1.688 | 7.36478E-08 | NCOA3 | -1.586 | 0.000334575 |
| RNF182 | 1.683 | 6.95903E-05 | CREBL2 | -1.582 | 1.4649E-06 |
| BHLHE40 | 1.682 | 7.18899E-08 | ANK3 | -1.577 | 0.0286206 |
| NPHP3 | 1.679 | 5.78736E-08 | PEX5 | -1.575 | 1.05635E-08 |
| GLDN | 1.679 | 3.24122E-05 | PLCG2 | -1.571 | 0.00294835 |
| PDCD4 | 1.672 | 7.94022E-05 | IL1RAP | -1.570 | 7.78577E-06 |
| PPFIBP1 | 1.667 | 0.00107143 | TMEM30B | -1.569 | 0.00670916 |
| ZNF181 | 1.666 | 6.00405E-05 | CCDC112 | -1.563 | 0.00014101 |
| EPC2 | 1.662 | 8.84859E-09 | NRIP1 | -1.563 | 8.22058E-06 |
| FOXO3 | 1.659 | 1.0925E-06 | GALNT3 | -1.562 | 0.0142515 |
| MAFF | 1.652 | 0.000616144 | CCDC91 | -1.562 | 2.61145E-06 |
| ZFP36L1 | 1.650 | 5.90042E-08 | RAD51AP1 | -1.556 | 0.000694202 |
| PIGV | 1.649 | 3.03304E-07 | FGD4 | -1.556 | 0.0387183 |
| TP53 | 1.647 | 0.00454155 | FAM175A | -1.554 | 0.000701569 |
| BCAT1 | 1.643 | 0.00018007 | ADD2 | -1.548 | 0.0116708 |
| PYROXD1 | 1.642 | 1.38148E-05 | WBP11P1 | -1.548 | 4.08654E-05 |
| PHLDB2 | 1.639 | 0.00176945 | RNF141 | -1.546 | 0.0016387 |
| **ROCK1** | 1.636 | 3.60439E-07 | PDLIM3 | -1.541 | 0.00237155 |
| KLF8 | 1.636 | 0.000304595 | KIAA1804 | -1.541 | 1.45143E-05 |
| PXDN | 1.628 | 2.48294E-08 | RBM47 | -1.538 | 0.00814146 |
| ZNF419 | 1.626 | 0.000398608 | DSC3 | -1.534 | 0.000538273 |
| PRDM1 | 1.622 | 0.0101347 | GCC2 | -1.534 | 0.0149327 |
| TMOD1 | 1.616 | 4.84092E-05 | RGPD1 | -1.534 | 0.0149327 |
| HKR1 | 1.612 | 5.90042E-08 | RGPD2 | -1.534 | 0.0149327 |
| R3HDM2 | 1.609 | 1.77105E-05 | RGPD5 | -1.534 | 0.0149327 |
| KIAA0430 | 1.607 | 0.00146165 | RGPD6 | -1.534 | 0.0149327 |
| DNER | 1.607 | 0.00536125 | PBX1 | -1.534 | 0.00936014 |
| DYNC2LI1 | 1.607 | 0.000475976 | PTBP3 | -1.531 | 2.32023E-05 |
| **Gene (PC-3)** | **Fold change** | **FDR p-value** | **Gene (RWPE1)** | **Fold change** | **FDR p-value** |
| IGF2BP2 | 1.604 | 6.4818E-07 | KIAA1467 | -1.531 | 0.000230437 |
| XPOT | 1.604 | 0.00232144 | TC2N | -1.524 | 0.0179713 |
| LONRF1 | 1.596 | 0.000245754 | KLHL12 | -1.524 | 7.355E-07 |
| FAM107B | 1.596 | 3.03217E-06 | TNFAIP3 | -1.524 | 0.0279977 |
| CARF | 1.592 | 0.000623467 | ME1 | -1.522 | 0.000165155 |
| HES1 | 1.589 | 2.12485E-06 | PDE3B | -1.515 | 0.0154048 |
| ZSWIM7 | 1.587 | 0.000131433 | FGF2 | -1.511 | 1.74995E-05 |
| TAOK3 | 1.586 | 0.000219542 | DYNC2LI1 | -1.511 | 1.74795E-05 |
| ZFP90 | 1.586 | 0.00105818 | ALDH5A1 | -1.510 | 0.0227977 |
| SPRED2 | 1.583 | 6.21026E-07 | CD302 | -1.510 | 0.00268932 |
| PTPRE | 1.583 | 0.00339778 | PTPN6 | -1.509 | 0.0014783 |
| ZNF226 | 1.578 | 6.01423E-05 | COPS7A | -1.507 | 2.56866E-07 |
| SPIN3 | 1.573 | 1.11769E-05 | TMEM159 | -1.506 | 0.00012316 |
| RHOQ | 1.566 | 3.95451E-05 | SLFN11 | -1.502 | 0.00434145 |
| KIAA1324L | 1.565 | 2.17046E-08 | ZBTB10 | -1.500 | 0.00582376 |
| ZNF441 | 1.563 | 7.85444E-05 | BCAT1 | -1.495 | 0.00201188 |
| SETDB2 | 1.562 | 0.000159881 | SSH2 | -1.495 | 0.00578515 |
| APOBEC3G | 1.561 | 0.00258586 | ZMYM2 | -1.493 | 0.0392845 |
| TRIM66 | 1.557 | 0.000130748 | PDE4A | -1.492 | 0.026846 |
| TADA3 | 1.555 | 0.000191881 | MED21 | -1.490 | 7.19014E-06 |
| C14orf28 | 1.552 | 0.00194013 | VASH2 | -1.488 | 0.0126166 |
| ID4 | 1.551 | 0.00597013 | GLUL | -1.488 | 0.0035494 |
| HOMER1 | 1.551 | 7.63847E-05 | USP13 | -1.486 | 1.22546E-06 |
| CYTL1 | 1.551 | 0.000077059 | NECAP1 | -1.484 | 1.25667E-05 |
| EIF2S2 | 1.550 | 3.5301E-08 | CD55 | -1.477 | 0.000690106 |
| PLEKHA5 | 1.549 | 2.66008E-06 | NR1D2 | -1.476 | 0.00662025 |
| ZNF236 | 1.548 | 0.0136996 | GNB4 | -1.474 | 0.00514805 |
| ZFAND3 | 1.548 | 8.68328E-08 | DERA | -1.472 | 0.00195873 |
| PRKAB2 | 1.546 | 5.22141E-06 | ABCC6P1 | -1.470 | 0.0130874 |
| MSR1 | 1.545 | 0.00208427 | BACH2 | -1.470 | 0.00186688 |
| KLF7 | 1.544 | 0.00627023 | GIMAP2 | -1.468 | 0.0354809 |
| TBC1D15 | 1.541 | 0.00648679 | CYYR1 | -1.464 | 6.78855E-05 |
| MITF | 1.540 | 0.00109782 | TMEM65 | -1.462 | 4.03716E-05 |
| ZNF655 | 1.537 | 0.000166965 | PMAIP1 | -1.462 | 0.0005131 |
| PCDHB13 | 1.531 | 0.0199503 | VAMP4 | -1.461 | 0.00130986 |
| N4BP2L2 | 1.531 | 0.000082265 | NPHP3 | -1.461 | 5.62031E-05 |
| SEC63 | 1.530 | 0.00195106 | NAMPT | -1.461 | 5.22618E-05 |
| VEZT | 1.529 | 0.000166038 | FTH1 | -1.454 | 0.000435513 |
| POPDC3 | 1.529 | 1.06398E-05 | MSL1 | -1.453 | 0.00015572 |
| NARS | 1.527 | 3.48941E-07 | THBS4 | -1.451 | 0.00978939 |
| GTDC1 | 1.525 | 0.00860934 | KIF14 | -1.450 | 0.00049908 |
| RCN1 | 1.517 | 1.9612E-08 | TFAP2A | -1.446 | 0.00912597 |
| BAZ2B | 1.514 | 8.74578E-05 | LPAR3 | -1.445 | 0.00535892 |
| CCDC112 | 1.512 | 0.000307642 | RPRD1A | -1.444 | 0.000630728 |
| GTF2H3 | 1.510 | 0.000341129 | TMEM183A | -1.444 | 7.94806E-05 |
| RCOR3 | 1.508 | 0.000629765 | TMEM183B | -1.444 | 7.94806E-05 |
| TRIM35 | 1.507 | 0.000211175 | ACBD3 | -1.444 | 0.00268713 |
| PPAPDC2 | 1.504 | 7.62127E-07 | PCMTD1 | -1.439 | 0.00289848 |
| MED13L | 1.504 | 2.69039E-05 | IPO7 | -1.438 | 2.82097E-06 |
| PID1 | 1.503 | 0.000441488 | PJA2 | -1.437 | 0.00183592 |
| **Gene (PC-3)** | **Fold change** | **FDR p-value** | **Gene (RWPE1)** | **Fold change** | **FDR p-value** |
| IGFBP3 | 1.502 | 0.000385316 | PHF21A | -1.433 | 0.000740608 |
| OGFRL1 | 1.502 | 0.00273358 | LYRM5 | -1.431 | 0.0351585 |
| ZNF561 | 1.498 | 6.81841E-05 | MIER3 | -1.430 | 0.0384077 |
| MXI1 | 1.497 | 0.000215502 | PHTF2 | -1.429 | 0.00363434 |
| FAM134B | 1.493 | 0.00908135 | SAT1 | -1.427 | 0.000470768 |
| GLS | 1.493 | 0.0287066 | CAMK2D | -1.424 | 0.000222006 |
| KHDRBS3 | 1.492 | 0.000123455 | ATP11B | -1.423 | 0.00049757 |
| GRPEL2 | 1.489 | 2.4792E-06 | STYK1 | -1.422 | 0.00198895 |
| MOSPD2 | 1.488 | 0.00115034 | CDK1 | -1.421 | 0.00118322 |
| CCDC82 | 1.487 | 0.0226023 | SBF2 | -1.419 | 0.00535987 |
| CDK19 | 1.487 | 0.00132859 | ZRANB1 | -1.418 | 0.00936295 |
| ZNF333 | 1.484 | 0.00471324 | ARAP2 | -1.417 | 0.0121372 |
| CTH | 1.483 | 1.14953E-05 | CTBS | -1.417 | 0.000229472 |
| C5 | 1.482 | 0.00419955 | AHR | -1.417 | 0.0117768 |
| ALCAM | 1.482 | 0.000060652 | AS3MT | -1.415 | 0.0183689 |
| FOXP1 | 1.481 | 1.74638E-07 | LMAN1 | -1.413 | 0.00648539 |
| PLAC8 | 1.481 | 0.0133279 | HOMER2 | -1.413 | 0.00518603 |
| SUV420H1 | 1.480 | 1.28507E-05 | KLHL7 | -1.411 | 0.00011393 |
| SLC8A1 | 1.480 | 0.017722 | MATR3 | -1.410 | 0.00120456 |
| DSP | 1.477 | 0.00178077 | ERRFI1 | -1.409 | 4.60686E-06 |
| SLC39A14 | 1.476 | 7.36478E-08 | ZIC5 | -1.408 | 0.0138526 |
| HOXB2 | 1.476 | 0.000338823 | WEE1 | -1.407 | 0.000677442 |
| PCMTD2 | 1.475 | 0.000103586 | OTUD1 | -1.407 | 0.0251443 |
| WDR5B | 1.474 | 0.00102724 | KRT222 | -1.406 | 0.0179402 |
| PJA1 | 1.473 | 1.28051E-07 | THAP2 | -1.405 | 0.00225194 |
| RBM18 | 1.473 | 8.19112E-05 | ARHGEF3 | -1.405 | 0.000290669 |
| ATG12 | 1.471 | 4.83597E-05 | KIF21A | -1.404 | 4.18871E-05 |
| SHISA2 | 1.469 | 0.00248673 | SCAMP1 | -1.403 | 0.000995467 |
| DCAF17 | 1.467 | 0.00813066 | GRIA3 | -1.401 | 0.0069217 |
| CEP57 | 1.466 | 0.00215468 | SEC24A | -1.401 | 0.00388762 |
| TTBK2 | 1.465 | 0.0128164 | ARHGAP12 | -1.399 | 0.00478374 |
| CHMP4C | 1.465 | 2.25275E-05 | ITPR2 | -1.396 | 0.00298872 |
| PLEKHF2 | 1.464 | 0.00411207 | BDH2 | -1.396 | 0.000221091 |
| TUFT1 | 1.463 | 0.000403146 | PGM3 | -1.396 | 8.97486E-06 |
| SGTB | 1.459 | 0.00659124 | AURKA | -1.394 | 0.00136366 |
| PCDH7 | 1.459 | 0.0200443 | TICAM2 | -1.394 | 0.000107649 |
| ZNF449 | 1.458 | 0.000176871 | TNKS2 | -1.394 | 0.00412295 |
| YPEL5 | 1.458 | 1.29967E-05 | GNA13 | -1.393 | 0.000799423 |
| ZNF740 | 1.457 | 0.00471055 | CDCA3 | -1.392 | 3.30499E-05 |
| SERPINE2 | 1.457 | 2.09419E-05 | NOTCH2 | -1.390 | 0.00720227 |
| FAM200B | 1.453 | 0.000138453 | GEMIN2 | -1.389 | 0.023168 |
| AZIN1 | 1.452 | 2.4522E-07 | MIB1 | -1.389 | 0.00728724 |
| FAM161A | 1.451 | 0.001355 | TNPO1 | -1.389 | 0.0213552 |
| DHFRL1 | 1.451 | 0.0383776 | ATAD1 | -1.389 | 0.000025539 |
| RIOK3 | 1.449 | 2.90869E-06 | TP63 | -1.387 | 0.000246331 |
| KLHDC1 | 1.448 | 0.0260301 | BCL6 | -1.387 | 0.0272466 |
| ZNF680 | 1.448 | 0.00175028 | IFFO1 | -1.386 | 0.012212 |
| ACVR1 | 1.446 | 0.000363513 | ARMC8 | -1.386 | 0.0192114 |
| PAN2 | 1.446 | 0.00016943 | MACC1 | -1.384 | 0.0198497 |
| PBX1 | 1.445 | 0.0128773 | CDC25C | -1.384 | 0.00022747 |
| **Gene (PC-3)** | **Fold change** | **FDR p-value** | **Gene (RWPE1)** | **Fold change** | **FDR p-value** |
| TNPO1 | 1.444 | 0.0080823 | PRKAA2 | -1.383 | 0.0153177 |
| SCAF8 | 1.443 | 3.08852E-05 | TIAL1 | -1.381 | 0.00423191 |
| ZNF227 | 1.441 | 0.000203277 | ICK | -1.380 | 0.00902129 |
| PSPH | 1.441 | 6.82747E-05 | PCDHA7 | -1.379 | 0.0339289 |
| DNAL4 | 1.439 | 0.00598926 | PCDHA1 | -1.379 | 0.0339289 |
| TRIM4 | 1.436 | 0.0118283 | PCDHA10 | -1.379 | 0.0339289 |
| RNF141 | 1.436 | 0.000491537 | PCDHA11 | -1.379 | 0.0339289 |
| ZNF302 | 1.434 | 0.00251634 | PCDHA12 | -1.379 | 0.0339289 |
| TRAPPC6B | 1.434 | 6.51075E-05 | PCDHA13 | -1.379 | 0.0339289 |
| IRX2 | 1.433 | 0.0270698 | PCDHA2 | -1.379 | 0.0339289 |
| XIAP | 1.431 | 0.000857397 | PCDHA3 | -1.379 | 0.0339289 |
| TSG101 | 1.429 | 2.8872E-07 | PCDHA4 | -1.379 | 0.0339289 |
| TRA2A | 1.428 | 0.0131832 | PCDHA5 | -1.379 | 0.0339289 |
| USP47 | 1.427 | 0.000259716 | PCDHA6 | -1.379 | 0.0339289 |
| FEZ2 | 1.426 | 0.000100303 | PCDHA8 | -1.379 | 0.0339289 |
| ZNF747 | 1.423 | 0.00109013 | PCDHAC1 | -1.379 | 0.0339289 |
| FAM120C | 1.422 | 0.00303593 | PCDHAC2 | -1.379 | 0.0339289 |
| LAMC2 | 1.422 | 0.000632202 | DNAJB9 | -1.378 | 0.0284859 |
| RBM39 | 1.420 | 5.74755E-06 | LAMA2 | -1.377 | 0.00879688 |
| MCL1 | 1.418 | 0.00407329 | TXNDC16 | -1.376 | 0.00104532 |
| FBXO11 | 1.417 | 1.08271E-06 | DEPDC1 | -1.376 | 0.00746611 |
| TGDS | 1.415 | 2.07453E-05 | STXBP3 | -1.375 | 0.0471385 |
| RNF170 | 1.412 | 0.0170259 | SATB1 | -1.375 | 0.0299208 |
| TNFRSF10B | 1.411 | 6.6388E-07 | GNAI1 | -1.374 | 0.00684932 |
| KIAA2026 | 1.410 | 0.000763469 | ZNF451 | -1.374 | 0.0196289 |
| TTPAL | 1.409 | 3.58308E-05 | USP47 | -1.372 | 1.34767E-05 |
| GPR37 | 1.407 | 0.00543863 | PRKCH | -1.372 | 0.000122234 |
| PRKCE | 1.406 | 2.06936E-05 | G2E3 | -1.372 | 0.0194064 |
| CD109 | 1.403 | 0.00026027 | PTPN2 | -1.370 | 2.20763E-05 |
| HNRNPA0 | 1.402 | 5.54665E-07 | WIPF1 | -1.369 | 0.018083 |
| CLK4 | 1.400 | 0.00997493 | SMC3 | -1.369 | 0.00151255 |
| RSRC2 | 1.399 | 0.0367451 | RERE | -1.368 | 0.00531044 |
| PDE8A | 1.399 | 5.73934E-05 | TGDS | -1.368 | 7.32066E-05 |
| L3MBTL4 | 1.395 | 0.0239612 | CSTA | -1.364 | 0.0374758 |
| TACSTD2 | 1.394 | 0.00267002 | AKR7A3 | -1.364 | 0.0379653 |
| AFTPH | 1.394 | 0.00104134 | PPP1R15B | -1.363 | 8.88094E-05 |
| ZNF90 | 1.394 | 0.020467 | KIAA0430 | -1.362 | 0.0336865 |
| HNRNPA2B1 | 1.393 | 0.000056764 | BAZ2B | -1.362 | 0.00012316 |
| RNF146 | 1.393 | 7.94022E-05 | SMAD4 | -1.361 | 5.35214E-05 |
| GGNBP2 | 1.392 | 5.35128E-08 | FBXO36 | -1.358 | 0.0212537 |
| PBLD | 1.392 | 0.00362445 | TMEM107 | -1.357 | 0.00312501 |
| INTU | 1.392 | 0.0275708 | SYNCRIP | -1.357 | 0.006025 |
| METTL6 | 1.391 | 0.00139896 | ANKRD12 | -1.357 | 0.00641097 |
| ZDBF2 | 1.391 | 0.0104733 | PDS5B | -1.357 | 0.00192264 |
| ING3 | 1.390 | 0.000048768 | USP32 | -1.355 | 0.000113408 |
| C2orf15 | 1.388 | 0.0316907 | FLRT3 | -1.355 | 0.0148985 |
| WDFY3 | 1.388 | 0.017946 | ATF7IP2 | -1.355 | 0.00275433 |
| SLC24A1 | 1.388 | 0.0258131 | ITCH | -1.354 | 0.00456922 |
| FLI1 | 1.387 | 0.0172042 | SUPT3H | -1.354 | 0.00278846 |
| DDX42 | 1.387 | 0.000205349 | FOXL2 | -1.354 | 0.0340771 |
| **Gene (PC-3)** | **Fold change** | **FDR p-value** | **Gene (RWPE1)** | **Fold change** | **FDR p-value** |
| TMEM43 | 1.385 | 4.99774E-05 | UBE2D1 | -1.351 | 0.000383377 |
| NR5A2 | 1.385 | 0.00733036 | GSDMA | -1.351 | 0.00901746 |
| SENP7 | 1.384 | 0.00770633 | MCC | -1.349 | 0.00126493 |
| FGF13 | 1.383 | 0.00150455 | PTK2 | -1.347 | 0.0013468 |
| IBTK | 1.383 | 0.00214414 | TPD52 | -1.347 | 0.000274199 |
| UGGT2 | 1.382 | 0.00423039 | SNX18 | -1.345 | 0.001975 |
| SBF2 | 1.382 | 0.00664438 | MPZL3 | -1.344 | 0.00788627 |
| EFNA1 | 1.380 | 0.000187132 | IFT81 | -1.344 | 0.00189699 |
| ZBTB21 | 1.380 | 0.000138869 | USP30 | -1.344 | 0.00656539 |
| ESCO1 | 1.379 | 0.0175983 | HERPUD2 | -1.343 | 0.00947862 |
| N4BP2L1 | 1.378 | 0.0102231 | TNFSF15 | -1.342 | 0.0142358 |
| HERPUD2 | 1.377 | 0.00867855 | MAP7 | -1.340 | 0.00283205 |
| ARHGAP42 | 1.376 | 0.00127959 | TOM1L1 | -1.339 | 0.000282089 |
| AMMECR1L | 1.376 | 2.57927E-05 | FOXO3 | -1.334 | 0.000490475 |
| BASP1 | 1.376 | 1.27538E-06 | PTEN | -1.333 | 0.00739189 |
| SOX4 | 1.374 | 0.000474751 | PEG10 | -1.333 | 0.0104618 |
| MCTP1 | 1.374 | 0.00434747 | MGEA5 | -1.332 | 0.00825673 |
| HSPA13 | 1.372 | 0.0150858 | FBXO8 | -1.331 | 0.000874591 |
| C9orf72 | 1.372 | 0.000439152 | TRPS1 | -1.331 | 0.0120649 |
| ZC3H7A | 1.371 | 0.000536722 | COG3 | -1.330 | 0.00354048 |
| TAS2R20 | 1.370 | 0.0303471 | HELZ | -1.330 | 0.0172141 |
| PDLIM5 | 1.370 | 0.0284067 | NEK7 | -1.329 | 0.00497104 |
| TLE1 | 1.370 | 0.000026643 | PUM2 | -1.328 | 0.0002589 |
| PHF3 | 1.370 | 0.00065411 | PDE4DIP | -1.324 | 0.0142487 |
| ITCH | 1.369 | 1.01652E-05 | FGF1 | -1.324 | 0.0147514 |
| TRIM9 | 1.368 | 0.0199537 | PRCP | -1.322 | 0.000420313 |
| AJUBA | 1.368 | 0.000356937 | ACAP2 | -1.322 | 0.00655158 |
| RNF139 | 1.367 | 1.45403E-07 | TACSTD2 | -1.321 | 0.0124371 |
| ACOT1 | 1.367 | 0.000287418 | SLC41A1 | -1.321 | 1.8359E-06 |
| ACOT2 | 1.367 | 0.000287418 | RBM27 | -1.319 | 0.000118946 |
| PTPN12 | 1.366 | 1.18575E-05 | GLRX | -1.319 | 0.0152927 |
| KLHL12 | 1.366 | 7.33141E-06 | NR2C1 | -1.318 | 0.00369269 |
| IL18R1 | 1.365 | 0.00959711 | WDR27 | -1.317 | 0.0394197 |
| KLF6 | 1.365 | 0.000434825 | MAP4K4 | -1.316 | 0.00301134 |
| MIDN | 1.364 | 0.000749101 | SNX16 | -1.316 | 0.0292283 |
| ADNP | 1.363 | 7.54983E-06 | TRAF3 | -1.316 | 0.000716817 |
| SCAMP1 | 1.363 | 0.00137478 | YAF2 | -1.316 | 0.0294078 |
| CGGBP1 | 1.358 | 0.000020654 | TRERF1 | -1.316 | 0.0120705 |
| KLF4 | 1.358 | 0.000780423 | CASK | -1.314 | 0.000497672 |
| ETV5 | 1.357 | 0.000286999 | IRAK2 | -1.311 | 0.0106011 |
| MEF2A | 1.356 | 0.000404349 | PRKCI | -1.309 | 0.0169304 |
| NCK1 | 1.356 | 9.06573E-05 | PTER | -1.307 | 0.0201735 |
| NEDD4L | 1.356 | 0.0271216 | PRDX3 | -1.307 | 4.08654E-05 |
| ZFHX4 | 1.355 | 0.0030501 | FBXW11 | -1.307 | 0.00256139 |
| NPC1 | 1.354 | 1.69056E-05 | DSCC1 | -1.306 | 0.00438434 |
| SH3RF1 | 1.354 | 0.00046617 | RNF138 | -1.306 | 0.016965 |
| BET1 | 1.353 | 0.00163898 | COG6 | -1.306 | 0.00223466 |
| ANKRD29 | 1.353 | 0.0269767 | M6PR | -1.304 | 0.000549839 |
| CREBBP | 1.353 | 0.0274311 | ULBP2 | -1.304 | 0.000529634 |
| SMG1 | 1.352 | 0.0313351 | CEP68 | -1.304 | 0.00830876 |
| **Gene (PC-3)** | **Fold change** | **FDR p-value** | **Gene (RWPE1)** | **Fold change** | **FDR p-value** |
| SLC11A2 | 1.350 | 0.00373799 | WASF2 | -1.303 | 0.0465217 |
| TMEM167B | 1.350 | 7.75265E-05 | DEPDC1B | -1.302 | 0.000135165 |
| SYS1 | 1.349 | 0.000701141 | MED13L | -1.302 | 0.00218459 |
| GORAB | 1.346 | 1.61966E-05 | LRRC8B | -1.301 | 0.00200125 |
| ZNF567 | 1.345 | 0.0262595 | SMARCA5 | -1.301 | 0.00542502 |
| COPG2 | 1.344 | 0.00312761 | CBWD5 | -1.301 | 0.0291897 |
| TRIM13 | 1.344 | 0.0044702 | SLC23A2 | -1.300 | 0.0368685 |
| SP3 | 1.343 | 0.0171495 | LSM14A | -1.300 | 0.00887578 |
| RBL2 | 1.342 | 0.000781759 | FAM133A | -1.300 | 0.0232302 |
| MERTK | 1.342 | 0.0294145 | XIAP | -1.299 | 0.0113517 |
| PTK2B | 1.342 | 0.0343848 | R3HDM2 | -1.298 | 0.00535573 |
| THBS1 | 1.342 | 0.0052978 | KIF1B | -1.298 | 0.0014614 |
| ANAPC16 | 1.342 | 0.000843561 | NUCKS1 | -1.298 | 0.000743907 |
| PPIL4 | 1.340 | 0.00269552 | ZCCHC14 | -1.297 | 6.02455E-06 |
| PCDHA7 | 1.340 | 0.010971 | KBTBD3 | -1.297 | 0.034855 |
| PCDHA1 | 1.340 | 0.010971 | CEP57 | -1.297 | 0.00954025 |
| PCDHA10 | 1.340 | 0.010971 | CERS6 | -1.296 | 0.00898367 |
| PCDHA11 | 1.340 | 0.010971 | PURB | -1.296 | 0.00375709 |
| PCDHA12 | 1.340 | 0.010971 | STK3 | -1.296 | 0.0287486 |
| PCDHA13 | 1.340 | 0.010971 | CLEC2B | -1.295 | 0.00341425 |
| PCDHA2 | 1.340 | 0.010971 | CSNK1G3 | -1.292 | 0.00604536 |
| PCDHA3 | 1.340 | 0.010971 | CPEB4 | -1.292 | 0.00396235 |
| PCDHA4 | 1.340 | 0.010971 | SETDB2 | -1.290 | 0.0174962 |
| PCDHA5 | 1.340 | 0.010971 | LIN9 | -1.289 | 0.0243325 |
| PCDHA6 | 1.340 | 0.010971 | PDCD4 | -1.289 | 0.024829 |
| PCDHA8 | 1.340 | 0.010971 | SLC39A6 | -1.287 | 0.00445449 |
| PCDHAC1 | 1.340 | 0.010971 | TMEM161B | -1.287 | 0.00700404 |
| PCDHAC2 | 1.340 | 0.010971 | XRCC4 | -1.286 | 0.0353789 |
| CSNK1A1 | 1.340 | 0.0294718 | TRIQK | -1.286 | 0.000384157 |
| PLOD2 | 1.340 | 3.35247E-05 | LHFPL2 | -1.285 | 0.0147472 |
| LARP1B | 1.338 | 0.0124785 | RCOR1 | -1.285 | 0.00225843 |
| RASA1 | 1.337 | 0.00894867 | ELOVL7 | -1.285 | 0.00705629 |
| ABCA5 | 1.337 | 0.00699314 | APIP | -1.284 | 2.38006E-05 |
| RPGR | 1.335 | 0.0271312 | GBAS | -1.283 | 0.0190979 |
| ASF1A | 1.335 | 0.0142374 | KMT2E | -1.282 | 0.0123757 |
| CDC42BPA | 1.334 | 0.0073831 | AGL | -1.282 | 0.0152226 |
| ZNF532 | 1.334 | 0.00199875 | ATP2B1 | -1.282 | 0.0319444 |
| USP25 | 1.333 | 0.00346852 | GM2A | -1.282 | 0.000110425 |
| KIF27 | 1.333 | 0.04338 | VEGFA | -1.281 | 0.000284127 |
| PSEN1 | 1.333 | 0.000033956 | PHC1 | -1.280 | 0.0114825 |
| IFT81 | 1.333 | 0.010316 | USP54 | -1.280 | 0.00535471 |
| DPY19L3 | 1.332 | 0.021969 | TDG | -1.279 | 0.0282559 |
| MYC | 1.332 | 4.45777E-05 | AGFG1 | -1.278 | 0.0362925 |
| EHBP1 | 1.331 | 0.02444 | HSPA13 | -1.278 | 0.00219297 |
| TM9SF1 | 1.331 | 5.69662E-05 | SHOC2 | -1.277 | 3.70942E-05 |
| ZNF215 | 1.326 | 0.0152925 | CEP350 | -1.277 | 0.000981894 |
| PTPN21 | 1.326 | 0.0171724 | RBBP5 | -1.277 | 0.000193005 |
| ZNF214 | 1.325 | 0.0293965 | OXR1 | -1.276 | 0.0492078 |
| LZTFL1 | 1.325 | 0.000187132 | TMTC2 | -1.276 | 0.0293555 |
| MRPS10 | 1.324 | 2.43618E-05 | PAFAH1B2 | -1.275 | 0.0474966 |
| **Gene (PC-3)** | **Fold change** | **FDR p-value** | **Gene (RWPE1)** | **Fold change** | **FDR p-value** |
| FBXO22 | 1.323 | 0.0348097 | KIAA1841 | -1.275 | 0.00578232 |
| SH3GL3 | 1.323 | 0.00760383 | VEZT | -1.275 | 0.0176755 |
| ZNF451 | 1.322 | 3.85909E-05 | EPHX2 | -1.274 | 0.04497 |
| ZNF592 | 1.321 | 0.00237943 | RBM15 | -1.274 | 0.0334826 |
| TMEM168 | 1.319 | 1.95117E-05 | NFKBIZ | -1.273 | 0.00725421 |
| ELP3 | 1.319 | 5.36292E-05 | CMTM4 | -1.273 | 0.0466513 |
| BACH1 | 1.318 | 0.00630856 | PDS5A | -1.272 | 0.00311992 |
| CDH13 | 1.318 | 0.000887167 | TBC1D12 | -1.272 | 0.00218459 |
| RNMT | 1.318 | 0.00653735 | IQCB1 | -1.272 | 0.000661528 |
| FNIP1 | 1.318 | 0.0053484 | BHLHE40 | -1.272 | 0.00035719 |
| CBWD5 | 1.318 | 0.0173699 | STAG2 | -1.271 | 0.00424035 |
| CSNK2A1 | 1.317 | 0.0431404 | CNOT6 | -1.270 | 0.00380131 |
| CDK14 | 1.317 | 0.0398981 | NR3C2 | -1.270 | 0.0132651 |
| TAF1B | 1.317 | 0.0153014 | TSPAN31 | -1.268 | 0.0120285 |
| PTPN2 | 1.316 | 0.0283681 | DARS | -1.267 | 0.0490935 |
| PHKA1 | 1.315 | 0.0211111 | UBR5 | -1.267 | 0.028863 |
| ANO6 | 1.315 | 0.0133778 | HIVEP2 | -1.267 | 0.00116481 |
| KLHDC10 | 1.313 | 0.0354495 | USP53 | -1.265 | 0.00277236 |
| TCP11L1 | 1.311 | 0.0243437 | SASS6 | -1.264 | 0.025433 |
| DUSP11 | 1.311 | 4.94321E-05 | AFTPH | -1.264 | 0.0172141 |
| PMAIP1 | 1.311 | 0.00502458 | HES1 | -1.263 | 0.00224466 |
| PDCD6IP | 1.310 | 0.00258045 | CPD | -1.262 | 0.00286147 |
| MFSD6 | 1.306 | 0.00502458 | NCAPH | -1.262 | 0.000291813 |
| KDM4C | 1.305 | 0.00071454 | NDUFS1 | -1.262 | 0.000601389 |
| CCDC91 | 1.305 | 0.000217953 | SEC24B | -1.262 | 0.0010641 |
| ATP11B | 1.304 | 0.00714216 | MARVELD3 | -1.262 | 0.0155697 |
| MOSPD1 | 1.304 | 6.41092E-05 | SEC24D | -1.261 | 0.0361608 |
| ARMCX5 | 1.304 | 0.000922246 | PEX2 | -1.261 | 0.00954287 |
| TGIF1 | 1.303 | 0.00278209 | SPCS3 | -1.260 | 0.000175436 |
| ARL13B | 1.302 | 0.00138422 | NAP1L1 | -1.258 | 0.0298388 |
| **PPM1B** | 1.301 | 0.0150408 | EIF2AK3 | -1.258 | 0.00543057 |
| KIAA1109 | 1.301 | 0.028949 | WASL | -1.258 | 0.0500723 |
| RBKS | 1.300 | 0.00258854 | PACRGL | -1.256 | 0.000400969 |
| ORC5 | 1.298 | 0.0068476 | SYS1 | -1.255 | 0.000395656 |
| PUS10 | 1.296 | 0.00782114 | ZNF720 | -1.254 | 0.0115165 |
| ZDHHC17 | 1.295 | 0.00126786 | ZFAND3 | -1.254 | 0.000663951 |
| MTX3 | 1.295 | 0.00609067 | CPEB3 | -1.254 | 0.0101288 |
| PSME4 | 1.295 | 0.00160888 | SP3 | -1.253 | 0.00293117 |
| EYA2 | 1.294 | 0.0352366 | KLHL31 | -1.253 | 0.00389996 |
| APPL2 | 1.294 | 0.00871666 | BAZ2A | -1.251 | 0.00322723 |
| MDK | 1.293 | 0.0154563 | UBE2K | -1.251 | 0.00598082 |
| ZNF786 | 1.293 | 0.0025377 | MAT2B | -1.250 | 0.030717 |
| XRN1 | 1.292 | 0.0307593 | CPAMD8 | -1.250 | 0.0127408 |
| CNOT4 | 1.290 | 0.00160565 | TYW3 | -1.249 | 0.00860701 |
| CLUAP1 | 1.290 | 0.00206715 | ATF2 | -1.248 | 0.0384991 |
| PAX6 | 1.286 | 0.0322295 | FBXO11 | -1.248 | 0.0240729 |
| MGEA5 | 1.286 | 0.0069168 | IBTK | -1.248 | 0.0337199 |
| RNF19A | 1.286 | 0.000821021 | **PTTG1** | -1.247 | 0.0010396 |
| KIF18A | 1.284 | 0.0384628 | TBL1XR1 | -1.245 | 0.00555471 |
| KMT2E | 1.284 | 0.0166024 | EDEM3 | -1.244 | 0.00916525 |
| **Gene (PC-3)** | **Fold change** | **FDR p-value** | **Gene (RWPE1)** | **Fold change** | **FDR p-value** |
| CTBS | 1.284 | 0.0024098 | FGF23 | -1.242 | 0.0318008 |
| UFM1 | 1.284 | 0.002226 | DDX21 | -1.242 | 0.0117432 |
| FBXO30 | 1.284 | 0.000202342 | KLHL15 | -1.242 | 0.0152199 |
| KIAA2018 | 1.283 | 0.000866991 | TAB2 | -1.242 | 0.00146004 |
| EFTUD1 | 1.283 | 8.14051E-05 | DCTN4 | -1.241 | 0.000067402 |
| LMBR1 | 1.282 | 7.10362E-05 | NLN | -1.241 | 0.0226765 |
| NIPSNAP3B | 1.282 | 0.0140806 | IRF2BP2 | -1.241 | 0.00274196 |
| C2orf68 | 1.282 | 0.00742724 | LRRC37B | -1.241 | 6.50076E-05 |
| SPIN1 | 1.282 | 8.74092E-06 | PHLDB2 | -1.241 | 0.0109091 |
| PACRGL | 1.279 | 0.00615268 | SPAG9 | -1.240 | 0.0415555 |
| CSNK1G3 | 1.278 | 0.00590006 | SRPK2 | -1.239 | 0.000301317 |
| ZNF790 | 1.277 | 0.0207966 | ZSWIM6 | -1.239 | 0.000201616 |
| UBXN2B | 1.277 | 0.00628236 | TAOK3 | -1.238 | 0.00114419 |
| INHBE | 1.276 | 0.0232467 | FOXJ3 | -1.238 | 8.68213E-05 |
| RYBP | 1.276 | 0.000366301 | KCNG3 | -1.238 | 0.0128491 |
| ATXN7 | 1.275 | 0.00351996 | SNX4 | -1.237 | 0.00215132 |
| LATS2 | 1.274 | 0.00026488 | NIPA1 | -1.237 | 0.0184718 |
| FOXL1 | 1.274 | 0.000564221 | FOXN2 | -1.236 | 0.00527498 |
| XPR1 | 1.273 | 0.000677376 | EIF4A2 | -1.235 | 0.00494459 |
| RB1CC1 | 1.273 | 0.00523944 | CAMTA1 | -1.235 | 0.00432915 |
| RAB5A | 1.273 | 1.53154E-05 | TRAPPC6B | -1.235 | 0.00128986 |
| ARFGAP2 | 1.271 | 0.00439178 | CTH | -1.235 | 0.00470295 |
| CBLL1 | 1.271 | 0.00215062 | ATG4C | -1.234 | 0.00185121 |
| DND1 | 1.270 | 0.0212042 | PGM2L1 | -1.234 | 0.0310403 |
| STK10 | 1.269 | 0.00537523 | ANXA7 | -1.233 | 0.0147709 |
| MSL2 | 1.269 | 0.00101711 | SRRM1 | -1.232 | 0.000979271 |
| KCTD15 | 1.268 | 0.00188263 | DNAJB14 | -1.232 | 0.0227738 |
| RABGEF1 | 1.268 | 0.0024932 | COQ10B | -1.232 | 0.0134748 |
| CD2BP2 | 1.267 | 0.00100626 | NRAS | -1.230 | 0.000804322 |
| SOS1 | 1.267 | 0.000444383 | NDRG3 | -1.230 | 0.000426837 |
| ZNF493 | 1.267 | 0.00980807 | FNDC3A | -1.230 | 0.0359448 |
| SCYL3 | 1.267 | 0.00110467 | SGMS1 | -1.230 | 0.0290458 |
| TYW1B | 1.265 | 0.000214994 | FAM171B | -1.230 | 0.0447859 |
| MED19 | 1.265 | 0.00063477 | FAM175B | -1.230 | 0.0105263 |
| PHF21A | 1.265 | 0.0117475 | MOSPD2 | -1.229 | 0.0258477 |
| DCTN4 | 1.264 | 1.69298E-05 | GRM5 | -1.229 | 0.022514 |
| TIA1 | 1.263 | 0.00956568 | ATG10 | -1.229 | 0.00330746 |
| LPGAT1 | 1.263 | 0.00266139 | PARG | -1.228 | 0.0137441 |
| ZNF28 | 1.263 | 0.0293965 | ASPH | -1.228 | 0.00670109 |
| ZNF468 | 1.263 | 0.0293965 | NCKAP1 | -1.228 | 0.00251788 |
| ARL8B | 1.262 | 3.15664E-05 | TAF4 | -1.227 | 4.22295E-05 |
| SLTM | 1.262 | 0.00099302 | RSF1 | -1.226 | 0.0174694 |
| SNAI2 | 1.261 | 0.00252137 | FYCO1 | -1.226 | 0.0405274 |
| ACVR2A | 1.261 | 0.0301961 | GALNT1 | -1.226 | 0.00268319 |
| SRGAP2 | 1.260 | 0.000414575 | XPO4 | -1.226 | 0.00819535 |
| NAB2 | 1.260 | 0.0117354 | HNRNPH3 | -1.225 | 0.00180204 |
| PPIL3 | 1.260 | 0.00483693 | ISCA1 | -1.225 | 0.0157269 |
| USP3 | 1.259 | 0.000729063 | ADORA1 | -1.224 | 0.0196043 |
| EBAG9 | 1.259 | 0.0344997 | ATAD2 | -1.224 | 0.00518603 |
| YEATS4 | 1.259 | 0.00513719 | MCF2L | -1.224 | 0.00197194 |
| **Gene (PC-3)** | **Fold change** | **FDR p-value** | **Gene (RWPE1)** | **Fold change** | **FDR p-value** |
| SUPT7L | 1.259 | 0.03063 | CWF19L2 | -1.223 | 0.00901977 |
| RCHY1 | 1.258 | 0.0335178 | TRIM13 | -1.222 | 0.0101844 |
| TRIB2 | 1.258 | 0.0120517 | MELK | -1.220 | 0.0205067 |
| VCPIP1 | 1.258 | 0.00188263 | BTRC | -1.219 | 0.00536177 |
| RABIF | 1.257 | 0.00800828 | TMEM38B | -1.219 | 0.000582783 |
| LGALS8 | 1.256 | 0.001703 | ZNF174 | -1.219 | 0.00747085 |
| ERP29 | 1.256 | 0.000853156 | C11orf58 | -1.219 | 0.0170819 |
| CRY1 | 1.256 | 0.034497 | KLF5 | -1.219 | 0.00996416 |
| NINL | 1.255 | 0.00716488 | PXMP4 | -1.218 | 0.0465095 |
| ARL4C | 1.255 | 0.00259619 | RPRD2 | -1.217 | 0.0215771 |
| UACA | 1.254 | 2.45586E-05 | YY1 | -1.217 | 0.0227431 |
| UBE3B | 1.254 | 0.00146362 | TMEM87B | -1.217 | 0.0200442 |
| ZNF706 | 1.254 | 0.0286435 | FAM45A | -1.216 | 0.0299511 |
| ZSCAN2 | 1.254 | 0.0127899 | FAM45B | -1.216 | 0.0299511 |
| LAMC1 | 1.254 | 0.00539102 | CA1 | -1.216 | 0.0272492 |
| LSM14A | 1.252 | 0.0370482 | PKP4 | -1.216 | 0.000799423 |
| GLI4 | 1.251 | 0.0423966 | MYC | -1.216 | 0.00213215 |
| ARHGEF3 | 1.251 | 0.00552827 | WAC | -1.216 | 0.00053001 |
| TRMT5 | 1.251 | 0.000111465 | ASCC1 | -1.215 | 0.00142937 |
| PHC1 | 1.251 | 0.0160399 | JARID2 | -1.214 | 0.0406636 |
| NAA25 | 1.250 | 0.0116816 | NAA15 | -1.214 | 0.00179042 |
| WAC | 1.249 | 9.71751E-05 | MIER1 | -1.214 | 0.0250355 |
| GABPA | 1.249 | 0.011802 | RSBN1L | -1.212 | 0.00557036 |
| HNRNPU | 1.249 | 2.41338E-05 | TOP1 | -1.212 | 0.037912 |
| OXNAD1 | 1.249 | 0.00141585 | PLEKHA1 | -1.211 | 0.043833 |
| RCOR1 | 1.248 | 0.00403662 | NIT2 | -1.211 | 0.000232612 |
| CDYL | 1.247 | 0.000394109 | PNN | -1.210 | 0.00819886 |
| ST7 | 1.245 | 0.0266913 | TGIF1 | -1.207 | 0.0329799 |
| C1QTNF3 | 1.245 | 0.0394925 | CAB39 | -1.207 | 3.83868E-05 |
| SIAH2 | 1.244 | 0.000854095 | TARBP1 | -1.206 | 0.00222027 |
| ZNF554 | 1.243 | 0.018595 | PPIC | -1.206 | 0.046377 |
| NUDT3 | 1.243 | 9.58768E-05 | PIGV | -1.206 | 0.00535892 |
| TMEM53 | 1.241 | 0.0269468 | TSG101 | -1.205 | 0.000376263 |
| CCDC144A | 1.241 | 0.00758895 | STYX | -1.204 | 0.00291597 |
| USP32 | 1.241 | 0.00758895 | SEC61A2 | -1.203 | 0.0479725 |
| SYNCRIP | 1.240 | 0.0369975 | EIF3A | -1.202 | 0.00300532 |
| FYCO1 | 1.239 | 0.0243954 | DIAPH2 | -1.202 | 0.0139349 |
| RAD50 | 1.239 | 0.00960018 | VPS26A | -1.202 | 0.0178265 |
| KLHL4 | 1.238 | 0.0395701 | GPBP1L1 | -1.202 | 0.00281146 |
| ATP8A1 | 1.238 | 0.0216181 | ZYG11B | -1.202 | 0.0412716 |
| PTPRJ | 1.237 | 0.0486254 | NDEL1 | -1.201 | 0.00646075 |
| SNUPN | 1.237 | 0.00179989 | BTF3 | -1.201 | 0.0330088 |
| WTAP | 1.237 | 0.000247195 | BUB3 | -1.200 | 0.00646075 |
| SSFA2 | 1.237 | 0.0289491 |  |  |  |
| GOLT1B | 1.234 | 0.0174125 |  |  |  |
| SEL1L | 1.234 | 0.000763511 |  |  |  |
| MLF1 | 1.233 | 0.0155849 |  |  |  |
| CDCA4 | 1.233 | 0.00202249 |  |  |  |
| ZNF20 | 1.232 | 0.0109112 |  |  |  |
| NKAP | 1.231 | 0.00183247 |  |  |  |
| **Gene (PC-3)** | **Fold change** | **FDR p-value** | **Gene (RWPE1)** | **Fold change** | **FDR p-value** |
| FOXN2 | 1.231 | 0.00432479 |  |  |  |
| ZNF319 | 1.231 | 0.00338845 |  |  |  |
| PAWR | 1.230 | 0.0134804 |  |  |  |
| TAPT1 | 1.230 | 0.00920679 |  |  |  |
| ZNF770 | 1.229 | 0.00318909 |  |  |  |
| PJA2 | 1.229 | 0.00295884 |  |  |  |
| GLRA2 | 1.229 | 0.0284256 |  |  |  |
| EXT1 | 1.226 | 0.000761941 |  |  |  |
| CREB3L4 | 1.224 | 0.0121826 |  |  |  |
| ESYT2 | 1.224 | 0.00472705 |  |  |  |
| AGFG1 | 1.224 | 0.0260219 |  |  |  |
| FOXD1 | 1.222 | 0.0256659 |  |  |  |
| NR2C1 | 1.221 | 0.0221435 |  |  |  |
| HPS5 | 1.220 | 0.000903075 |  |  |  |
| MAPK1IP1L | 1.220 | 0.000035477 |  |  |  |
| LIMS1 | 1.219 | 0.0021234 |  |  |  |
| UBA5 | 1.219 | 0.0359035 |  |  |  |
| APTX | 1.219 | 0.000325529 |  |  |  |
| CHD9 | 1.218 | 0.00307353 |  |  |  |
| KIAA0355 | 1.217 | 0.0046805 |  |  |  |
| CHST10 | 1.217 | 0.0036746 |  |  |  |
| TERF1 | 1.217 | 0.0258975 |  |  |  |
| ANKIB1 | 1.217 | 0.0330347 |  |  |  |
| YTHDC1 | 1.215 | 0.00107641 |  |  |  |
| RBM28 | 1.215 | 0.0049983 |  |  |  |
| CARKD | 1.214 | 3.14305E-05 |  |  |  |
| ACTR10 | 1.214 | 0.00496581 |  |  |  |
| FBLIM1 | 1.214 | 0.00193521 |  |  |  |
| MED23 | 1.213 | 0.026457 |  |  |  |
| C1orf52 | 1.213 | 0.000102677 |  |  |  |
| TRPC1 | 1.213 | 0.0165108 |  |  |  |
| OXR1 | 1.212 | 0.0187385 |  |  |  |
| C3orf33 | 1.211 | 0.0199219 |  |  |  |
| ZC3H15 | 1.211 | 0.00116012 |  |  |  |
| CSNK1E | 1.208 | 0.000552378 |  |  |  |
| MAN2A1 | 1.208 | 0.0159757 |  |  |  |
| CASK | 1.208 | 0.0318449 |  |  |  |
| RPRD2 | 1.207 | 0.0218327 |  |  |  |
| SUPT3H | 1.206 | 0.0414023 |  |  |  |
| BRD4 | 1.205 | 0.0367999 |  |  |  |
| EP300 | 1.205 | 0.0291805 |  |  |  |
| ASPH | 1.205 | 0.0490843 |  |  |  |
| FAM76B | 1.204 | 0.0120041 |  |  |  |
| FAM134A | 1.204 | 0.0113597 |  |  |  |
| ZNF460 | 1.203 | 0.00879303 |  |  |  |
| FAM13B | 1.203 | 0.0144665 |  |  |  |
| NSUN4 | 1.202 | 0.0232946 |  |  |  |
| SUMF2 | 1.202 | 4.51598E-05 |  |  |  |

**Additional File 4: Table S3. Aberrant gene expression in miR-186 inhibited PC-3 and miR-186 overexpressing RWPE1 cells.** Microarray gene list was restricted to genes down-regulated in RWPE1 (fold change ≤ -1.2) and up-regulated in PC-3 cells (fold change ≥ 1.2). Analysis revealed a down-regulation of 493 transcripts in RWPE1 cells and up-regulation of 547 transcripts in PC-3 cells. Genes in bold represent previously validated miR-186 targets. Moreover, genes were highlighted gray if modified in both RWPE1 with ectopic expression of miR-186 and miR-186 inhibited PC-3 cells.
